# Effects of resistance training on muscle mass, strength, and physical function in older women with sarcopenia: a systematic review and meta-analysis

**DOI:** 10.3389/fpubh.2025.1735899

**Published:** 2026-01-26

**Authors:** Ying Zhou, Kaiming Wen, Xinxin Zhang, Yulong Sun

**Affiliations:** 1 College of Physical Education and Health, Guangxi Normal University, Guilin, China; 2 Hainan Medical University, Haikou, China; 3 Shaanxi Normal University, Xi'an, China; 4 Guangxi University, Nanning, China

**Keywords:** muscle strength, older women, physical function, resistance training, sarcopenia

## Abstract

**Background and objective:**

Resistance training is widely recommended for sarcopenia, yet evidence in older women remains limited. This study systematically evaluated its effects on muscle mass, strength, and physical function in this population.

**Methods:**

PubMed, Embase, Web of Science, and the Cochrane Central Register of Controlled Trials were searched up to February 2025; the protocol was registered in PROSPERO (CRD420251066233). Meta-analyses were performed using the “meta” package in R under a random-effects model with restricted maximum likelihood estimation. Study quality was assessed using RoB 2, and evidence certainty via GRADE.

**Results:**

Twelve randomized controlled trials involving 518 older women with sarcopenia were included. Resistance training significantly improved handgrip strength (SMD = 0.43, 95% CI: 0.11–0.74), gait speed (SMD = 0.37, 95% CI: 0.09 to 0.64), knee extension strength (SMD = 0.85, 95% CI: 0.37 to 1.33), Timed Up and Go (SMD = −0.68, 95% CI: −0.95 to −0.41), and 30-Second Chair Stand (SMD = 0.52, 95% CI: 0.19 to 0.86), but had no significant effect on skeletal muscle mass index. Subgroup analyses showed that women with sarcopenic obesity achieved greater gains in knee extension strength, while those with sarcopenia alone improved more in gait speed. Mixed resistance training demonstrated a significant advantage in enhancing gait speed.

**Conclusion:**

Resistance training effectively enhances muscle strength and physical function in older women with sarcopenia, though improvements in muscle mass remain uncertain. Further large-scale, long-term studies are needed to confirm these findings and optimize intervention protocols.

## Graphical abstract

**Figure:**
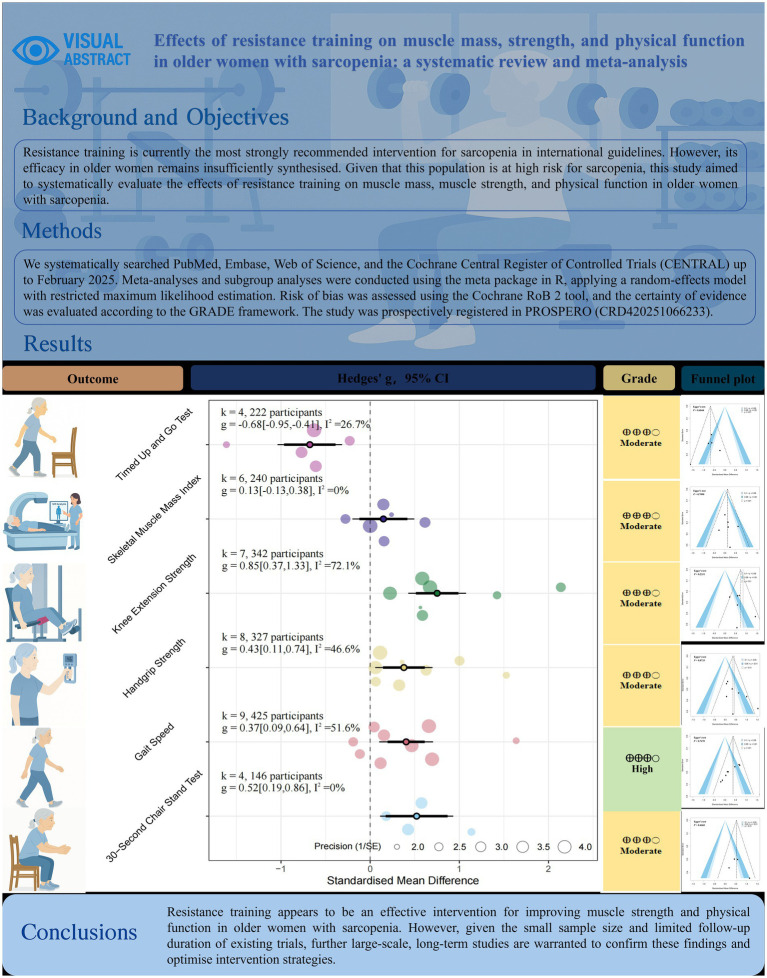

## 1 Introduction

Sarcopenia, defined as an age-related decline in skeletal muscle mass and function, has been firmly associated with adverse health outcomes, including functional impairment, mobility limitations, increased risks of falls and fractures, prolonged hospitalization, and elevated mortality rates (1, 2). Its prevalence varies widely depending on age, sex, ethnicity, and diagnostic criteria (3–5). With the rapid progression of the global population aging, the burden of sarcopenia is expected to rise further, imposing substantial pressures on healthcare systems and contributing to escalating medical costs (6).

A growing body of evidence suggests that women are at higher risk of sarcopenia than men (7–11). A recent nationwide cohort study confirmed that female sex remains an independent risk factor for sarcopenia, with women exhibiting approximately 50% higher odds of diagnosis even after adjusting for age, nutritional status, and chronic disease (12). This sex-based vulnerability may be attributed to lower baseline muscle mass, more pronounced age-related lean mass decline, and abrupt estrogen withdrawal following menopause (13, 14). In addition, a sedentary lifestyle, which is a known behavioral risk factor for sarcopenia, is more common among older women and may potentially exacerbate muscle loss and increase the risk of mortality (15, 16). These findings highlight the increased susceptibility of older women to sarcopenia and the urgent need for tailored intervention strategies.

The primary therapeutic goal for sarcopenia is to reverse or at least stabilize the decline in muscle strength while preserving or enhancing muscle mass to support functional independence and quality of life (17). Current international clinical guidelines, including those by the International Conference on Frailty and Sarcopenia Research (ICFSR), strongly recommend resistance training (RT) as the first-line intervention (18). Robust evidence indicates that RT can effectively improve muscle strength, mass, and functional performance through enhanced muscle protein synthesis and neuromuscular activation (17). However, most existing evidence is derived from mixed-gender populations, and systematic evaluations specifically targeting older women with clinically diagnosed sarcopenia remain limited (19, 20).

Recent meta-analyses have examined the preventive role of exercise in middle-aged, healthy women and demonstrated that early intervention could significantly delay declines in muscle mass, strength, and function (21, 22). However, these studies primarily included women younger than 65 years without sarcopenia, differing substantially from the high-risk population encountered in clinical settings. Compared to men or healthy individuals, older women with diagnosed sarcopenia often present with more complex pathophysiological features, including greater muscle loss, hormonal imbalances, and functional impairment (13, 14, 23). Evidence also suggests that older women exhibit blunted adaptive responses to RT regarding absolute gains in muscle strength and mass compared to older men (24, 25). In light of these gaps, high-quality intervention studies focusing specifically on older women with sarcopenia are urgently needed. The effectiveness and response patterns of RT in this vulnerable group remain unclear. Therefore, the present study aimed to systematically evaluate the effects of resistance training on muscle strength, muscle mass, and physical function in older women with sarcopenia, providing updated evidence to inform clinical decision-making and personalized management strategies.

## 2 Methods

### 2.1 Protocol and registration

The protocol of this systematic review and meta-analysis was registered in PROSPERO (CRD420251066233). This study was conducted in accordance with the PRISMA 2020 (Preferred Reporting Items for Systematic Reviews and Meta-Analyses) guidelines (26).

### 2.2 Search strategy and study selection

A comprehensive literature search was conducted in PubMed, Embase, Cochrane Library, and Web of Science, using MeSH terms and free-text keywords. The core search strategy included the following terms: (“Female”[MeSH Terms] OR “Women”[MeSH Terms]) AND (“Sarcopenia”[MeSH Terms]) AND (“Resistance training”[MeSH Terms]) AND ((“Older”[Title/Abstract]) OR (“Aged”[MeSH Terms]) OR (“Elderly”[Title/Abstract])). The full search strings for each database are provided in Appendix 1. Three independent reviewers (YZ, MK, and XX) screened the records and assessed eligibility. Disagreements were resolved through consultation with a fourth reviewer (YL). Reference lists of included articles and relevant systematic reviews were manually searched for additional eligible studies.

### 2.3 Eligibility criteria

We applied the PICOS framework (Participants, Interventions, Comparators, Outcomes, and Study Design) to determine study eligibility (27). Studies were included if they met all of the following criteria: (1) participants were females aged ≥ 60 years with sarcopenia, diagnosed according to any standard criteria or author-defined definitions that included at least one of the following: low muscle mass, low muscle strength, or impaired physical performance; (2) the intervention involved any form of resistance training, regardless of specific modality, intensity, or equipment used; (3) the comparator was usual care, health education, or no intervention; (4) outcomes were consistent with recommendations from the ICFSR, including Primary outcomes: handgrip strength, usual gait speed, skeletal muscle mass index (SMI); Secondary outcomes: knee extension strength, 30-s chair stand test (CST), and Timed Up and Go test (TUG); (5) the study was a randomized controlled trial (RCT).

Studies were excluded if they met any of the following criteria: (1) participants had specific comorbidities such as cancer, diabetes, stroke, HIV/AIDS, COPD, chronic kidney disease, liver cirrhosis, or had recently undergone organ transplantation; (2) interventions included drug therapy or nutritional supplementation; (3) conference abstracts, protocols, or systematic reviews; (4) non-English publications; (5) studies with insufficient data; or (6) full text unavailable through databases or direct contact with authors.

### 2.4 Data extraction

Two reviewers (YZ and YL) independently extracted the following information using a pre-defined data extraction form: study characteristics (first author, year of publication, country, setting, diagnostic criteria), participant characteristics (age, sample size), intervention details (duration, frequency, period), and outcome data (means and standard deviations for continuous outcomes, event rates for dichotomous outcomes). A third reviewer (MK) verified the extracted data. For studies with missing data, the corresponding author was contacted up to three times within 3 weeks.

### 2.5 Measures of treatment effect

For each outcome, mean differences (MD) and standard deviations (SD) of change from baseline were used to estimate treatment effects. When SD were not directly reported, they were calculated from standard errors, 95% confidence intervals, *p*-values, or t-statistics (28). If pre-post change SD were missing, they were imputed using the following formula:


$$SD_{\text{change}}=\sqrt{SD_{\text{baseline}}^{2}+SD_{\text{Post}}^{2}-2\times r\times SD_{\text{baseline}}\times SD_{\text{post}}}$$

In this formula, the SD of the change from baseline was imputed, assuming a correlation coefficient of 0.5 between pre- and post-intervention values (28). We acknowledge that this represents a conservative estimate, as physical function tests typically demonstrate high test–retest reliability. Consequently, this approach may overestimate the variance of change scores, resulting in wider confidence intervals and more conservative pooled estimates.

### 2.6 Quality assessment of evidence

We used the Cochrane Risk of Bias tool for randomized trials (RoB 2.0) to assess the methodological quality of each included study, evaluating the following domains: random sequence generation, allocation concealment, blinding, incomplete outcome data, and selective reporting (29). A study was rated as low risk of bias if all domains were judged to be low risk, high risk if at least one domain was considered high risk, and “some concerns” in other scenarios. Two independent reviewers conducted the assessments, with discrepancies resolved through discussion.

The certainty of evidence was evaluated using the GRADE approach, implemented via the GRADEpro GDT online tool^1^. Five domains were considered: risk of bias, inconsistency, indirectness, imprecision, and publication bias. The quality of evidence for each outcome was graded as “high,” “moderate,” “low,” or “very low” based on overall confidence in the effect estimate (30). Two reviewers independently conducted all assessments, with disagreements resolved by consensus. In addition, to assess potential small-study effects and publication bias, we constructed funnel plots and conducted visual inspections for each direct comparison.

### 2.7 Statistical analysis

Meta-analyses and subgroup analyses were conducted using the meta package in R software (version 4.3.1) (31). Random-effect models were fitted using the restricted maximum likelihood estimator (REML) to ensure robust estimation of pooled effect sizes (32). Given the small sample sizes of some included trials and moderate to high heterogeneity, the Hartung-Knapp adjustment was applied to refine confidence intervals and improve statistical precision (33). Between-group differences were evaluated using t-tests, with statistical significance at *p* < 0.05 (33).

To assess the reproducibility of findings in future similar studies, we calculated 95% prediction intervals (PI) for each outcome (34). If the PI fell entirely on one side of the null effect, the intervention effect was considered likely consistent; if it crossed the null, the future effect was deemed uncertain. We used inverse-variance weighting for continuous outcomes and reported pooled SMD with a corresponding 95% confidence interval (CI). Where possible, post-intervention endpoint data were prioritized; however, change-from-baseline values were also accepted when available. To correct for potential small-sample bias, Hedges’ g was used to estimate the effect size, with thresholds defined as small (g = 0.2), moderate (0.5), large (0.8), and very large (≥1.2) (35).

Statistical heterogeneity was assessed using the *I*^2^ statistic, with the following interpretation: < 40% (low), 40–75% (moderate), and >75% (high) (36). In addition to visual inspection of funnel plots, we performed Egger’s regression test to identify potential publication bias (37). Two sensitivity analyses were undertaken to examine the robustness of findings: (1) a leave-one-out analysis to evaluate the influence of each study on the pooled effect size and heterogeneity and (2) a comparison of fixed-effect and random-effects models to test the sensitivity of pooled estimates to model choice (38).

Subgroup analyses explored potential effect modifiers for outcomes exhibiting substantial heterogeneity. Subgroup analyses were stratified by: setting (institution-based vs. community-based), type of sarcopenia (sarcopenia vs. sarcopenic obesity), and type of resistance training (variable resistance, constant resistance, and mixed resistance). A *p*-value < 0.1 for interaction was considered statistically significant for subgroup effects.

## 3 Results

### 3.1 Literature selection and study characteristics

**Figure 1:**
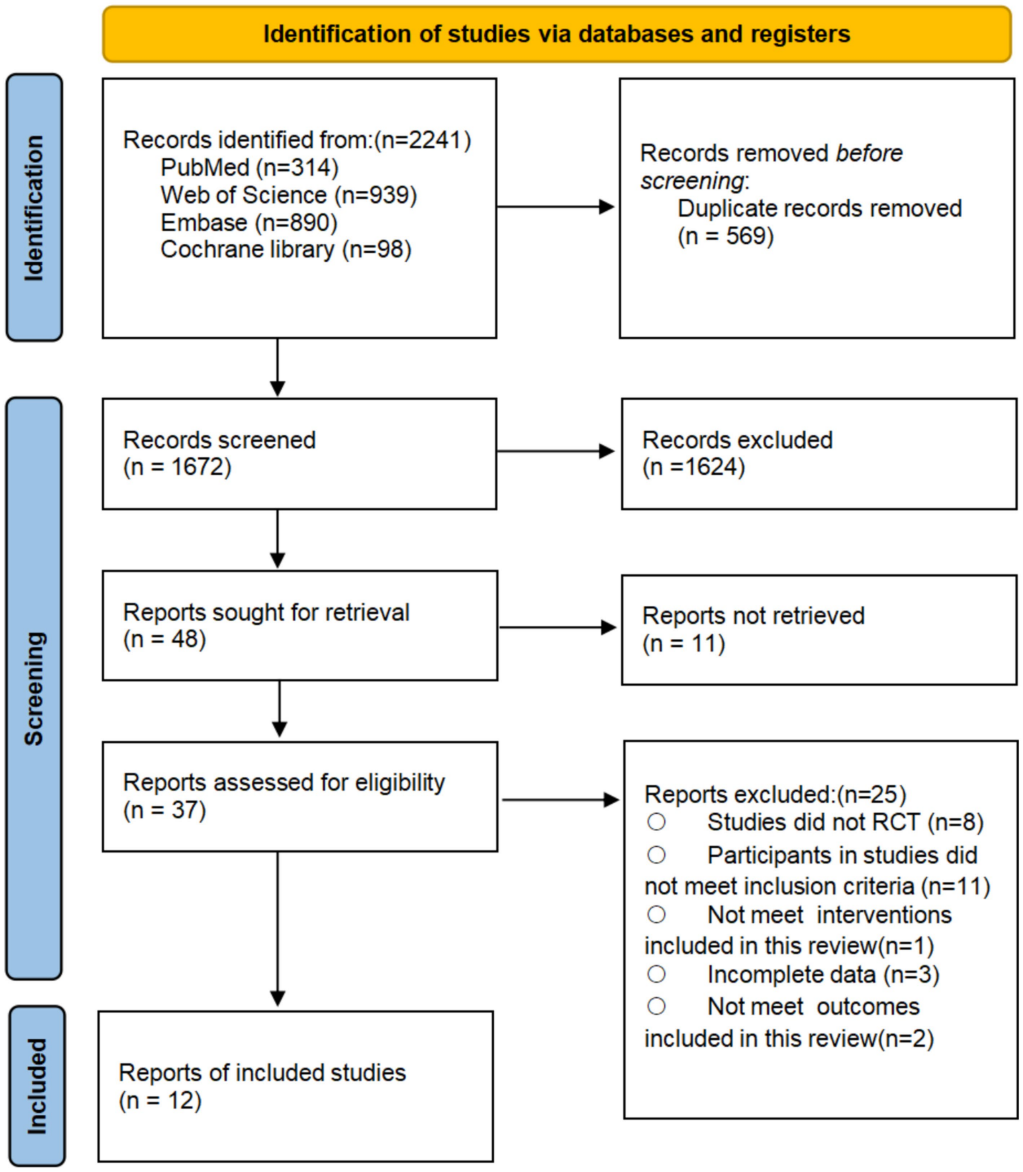
PRISMA flow diagram of the search process for studies.

Through systematic searches, 2,241 potentially relevant records were identified. After removing duplicates, 48 studies remained for the title and abstract screening. Following a full-text review of 37 articles, 12 RCTs were included in this review and meta-analysis, comprising 518 participants with a mean age of 72.38 ± 5.73 years. These trials were conducted across five countries, with the majority originating from China (*n* = 5), followed by Japan (*n* = 4), and one study each from Spain, Korea, and Brazil. The detailed screening and selection process is shown in Figure 1, and the characteristics of the included studies are presented in Table 1.

**Table 1:** Basic characteristics of the included studies.

Study ID	Intervention	Sample size	Age (Mean ± SD)	Frequency (times/week)	Duration (min)	Period (weeks)	Country	Diagnostic criteria	Setting	Types of sarcopenia
Huang et al. (2017)(67)	RT	18	68.89 ± 4.91	3	55	12	China	Study-defined criteria	Community	Sarcopenic Obesity
	CG	17	68.89 ± 4.91							
Lee et al. (2021) (68)	RT	15	70.13 ± 4.51	3	55	12	China	EWGSOP 2010	Community	Sarcopenic Obesity
	CG	12	71.82 ± 5.33							
Liao et al. (2017) (40)	RT	25	68.42 ± 5.86	3	60	12	China	EWGSOP 2010	Institution	Sarcopenic Obesity
	CG	21	66.39 ± 4.49							
Osuka et al. (2021) (43)	RT	38	71.8 ± 4.1	2	60	12	Japan	AWGS 2014	Institution	Sarcopenic
	CG	38	71.6 ± 4.2							
Rufino et al. (2023) (41)	RT	20	79.9 ± 7.2	2	65	26	Spain	EWGSOP 2010	Community	Sarcopenic
	CG	18	79.6 ± 7.7							
Seo et al. (2021) (39)	RT	12	70.3 ± 5.38	3	60	16	Korea	EWGSOP 2010	Institution	Sarcopenic
	CG	10	72.9 ± 4.75							
Vasconcelos et al. (2016) (42)	RT	14	72 ± 4.6	2	60	10	Brazil	Study-defined criteria	Community	Sarcopenic Obesity
	CG	14	72 ± 3.6							
Hamaguchi (2017) (69)	RT	7	60.4 ± 2.7	2	60	6	Japan	EWGSOP 2010	Community	Sarcopenic
	CG	8	60.6 ± 2.3							
Liao et al. (2018) (70)	RT	33	66.67 ± 4.54	3	55	12	China	Study-defined criteria	Institution	Sarcopenic Obesity
	CG	23	68.32 ± 6.05							
Chen et al. (2018) (71)	RT	17	66.7 ± 5.3	2	60	8	China	AWGS 2014	Community	Sarcopenic
	CG	16	68.3 ± 2.8							
Kim et al. (2012) (72)	RT	39	79.0 ± 2.9	2	60	12	Japan	Study-defined criteria	Community	Sarcopenic
	CG	39	78.7 ± 2.8							
Kim et al. (2012) (73)	RT	32	79.6 ± 4.2	2	60	12	Japan	Study-defined criteria	Community	Sarcopenic
	CG	32	80.2 ± 5.6							

### 3.2 Risk of bias and certainty of evidence

**Figure 2:**
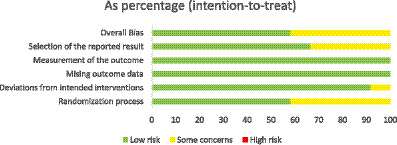
Overall risk of bias presented as a percentage of each risk of bias item across all included studies.

The risk of bias for each trial is presented in Figure 2. Overall, seven studies (58.3%) were judged to have a low risk of bias, while five studies (37.5%) were rated as having “some concerns.” All studies reported the generation of random sequences; however, three did not mention allocation concealment and were therefore rated as “unclear” for this domain. In selective outcome reporting, three studies lacked information on prespecified analysis protocols and did not register their protocols, resulting in “unclear risk of bias” ratings (Supplementary Table S2.1).

According to the GRADE framework, the certainty of evidence for all outcomes was rated as moderate, primarily due to concerns regarding potential bias in some studies and inter-study heterogeneity (Table 2).

**Table 2:** GRADE summary of evidence.

Outcome	Number of studies	Study design	Risk of bias	Inconsistency	Indirectness	Imprecision	Other consideration	Confidence rate
Handgrip strength	5	Randomized trials	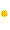	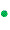	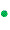	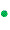	None	Moderate
Knee extension strength	5	Randomized trials	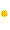	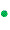	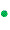	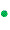	None	Moderate
Skeletal muscle mass index	5	Randomized trials	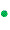	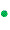	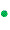	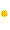	None	Moderate
Gait speed	6	Randomized trials	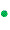	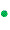	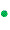	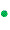	None	High
Timed Up and Go test	4	Randomized trials	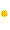	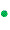	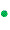	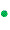	None	Moderate
30-Second chair stand test	3	Randomized trials	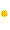	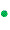	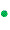	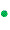	None	Moderate

Risk of bias: 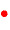, very serious; 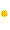, serious; 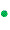, not serious.

### 3.3 Primary outcomes

**Figure 3:**
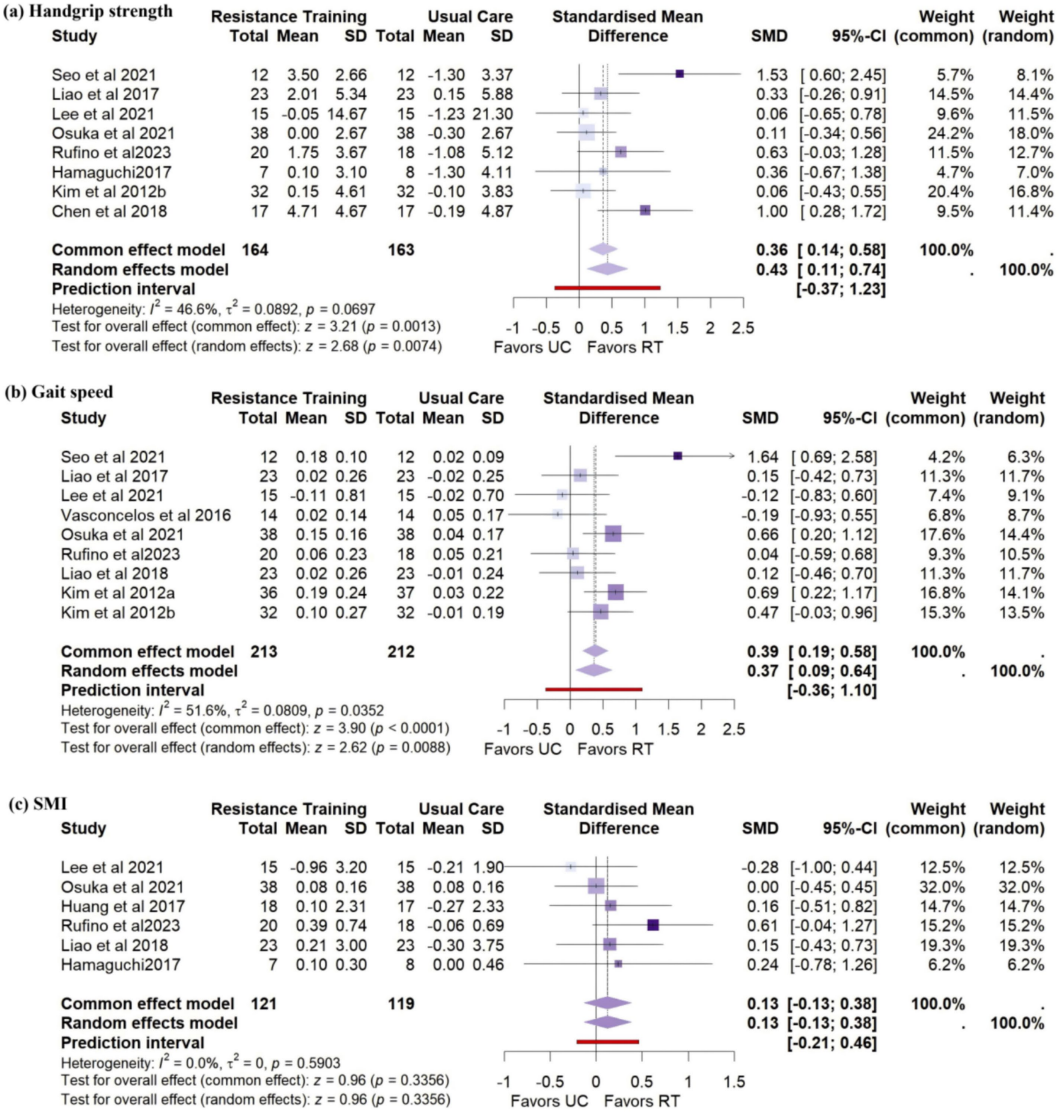
Forest plots of primary outcomes: **(a)** Handgrip strength, **(b)** Gait speed, and **(c)** SMI.

Eight studies (*n* = 327) reported changes in handgrip strength. Meta-analysis showed that RT significantly improved handgrip strength in older women with sarcopenia (SMD = 0.43, 95% CI: 0.11 to 0.74), with moderate heterogeneity (*I*^2^ = 46.6%) (Figure 3a).

Nine studies (*n* = 425) reported gait speed. The pooled analysis revealed statistically significant improvement (SMD = 0.37, 95% CI: 0.09 to 0.64), with moderate heterogeneity (*I*^2^ = 51.6%) (Figure 3b).

Six studies (*n* = 240) reported SMI. No significant improvement was observed (SMD = 0.13, 95% CI: −0.13 to 0.0.38), and heterogeneity was low (*I*^2^ = 0%) (Figure 3c).

### 3.4 Secondary outcomes

**Figure 4:**
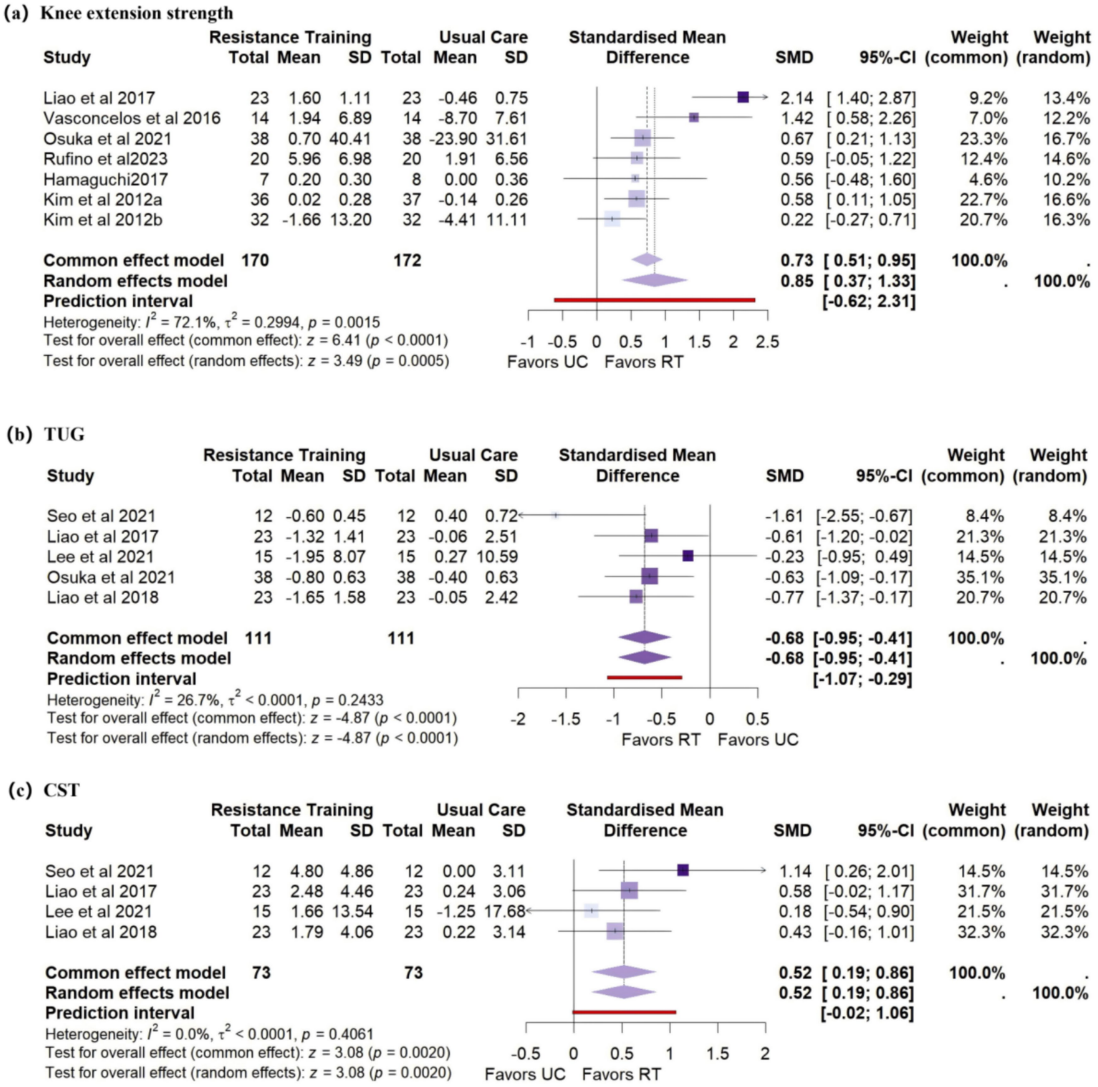
Forest plots of secondary outcomes: **(a)** Knee extension strength, **(b)** Timed Up and Go test, and **(c)** 30-s chair stand test.

Seven studies (*n* = 342) assessed changes in knee extension strength. Meta-analysis showed significant improvement following RT (SMD = 0.85, 95% CI: 0.37 to 1.33), with moderate heterogeneity (*I*^2^ = 72.1%) (Figure 4a).

Four studies (n = 222) reported TUG. The pooled estimate showed that RT significantly improved TUG performance (SMD = −0.68, 95% CI: −0.95 to −0.41), with low heterogeneity (I^2^ = 26.7%) (Figure 4b).

Four studies (n = 146) reported CST. RT had a statistically significant positive effect on CST performance (SMD = 0.52, 95% CI: 0.19 to 0.86), with low heterogeneity (I^2^ = 0%) (Figure 4c).

### 3.5 Subgroup analysis

**Figure 5:**
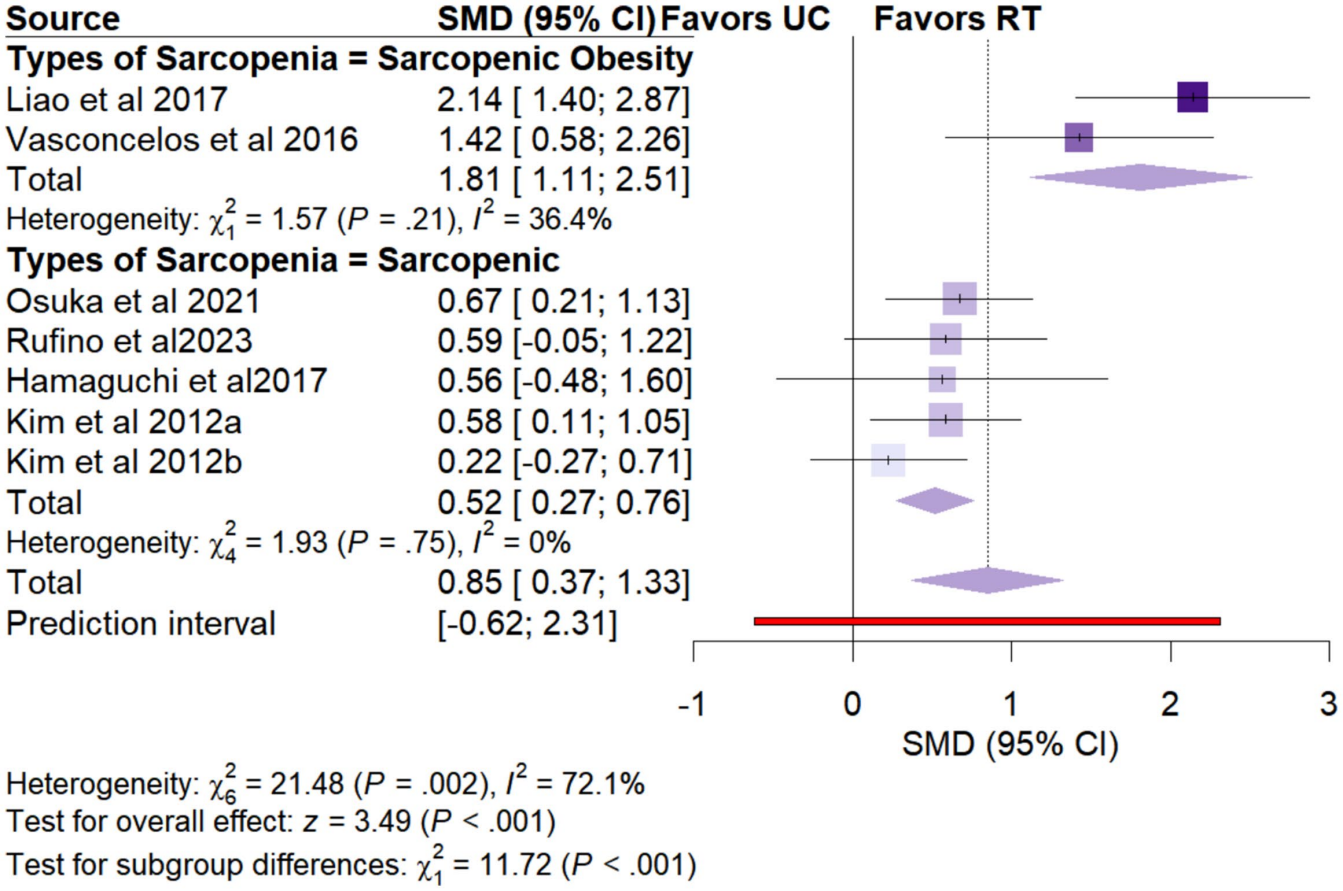
Forest plot of subgroup analyses for knee extension strength.

**Figure 6:**
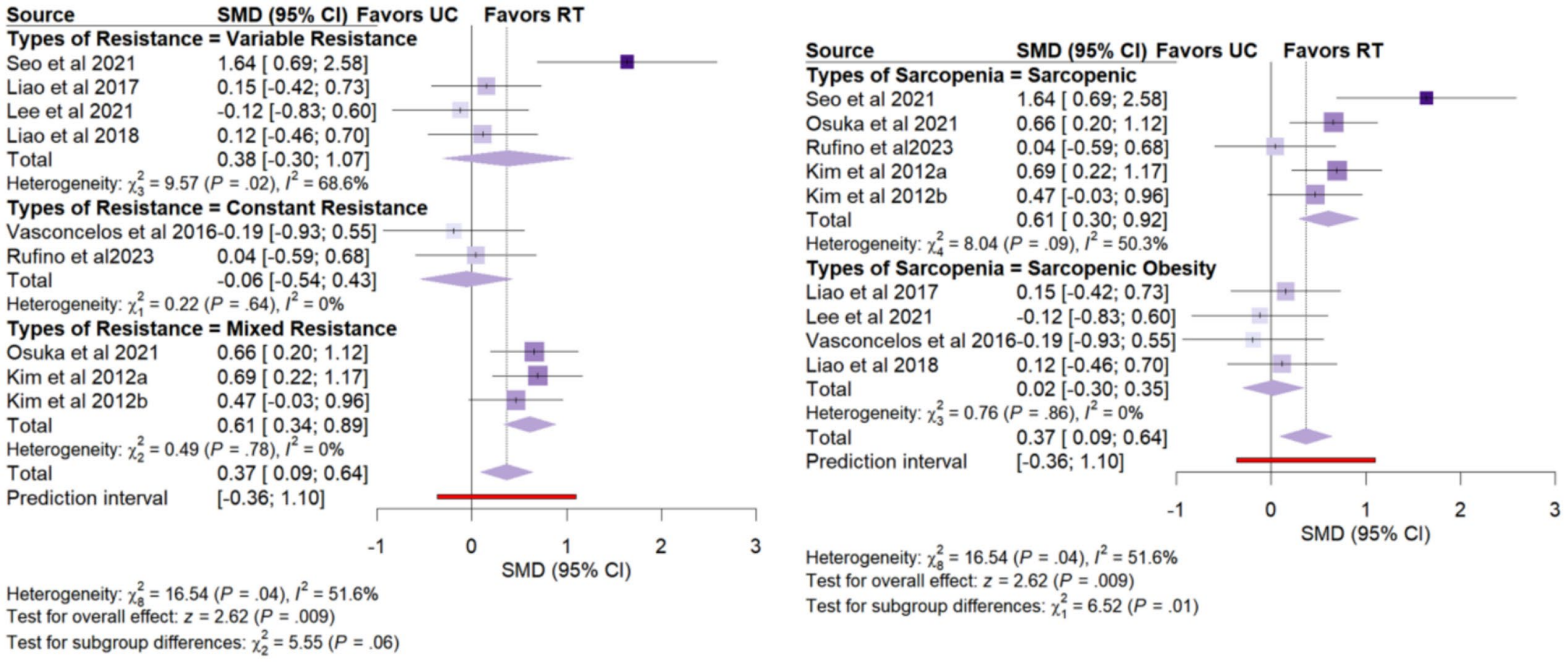
Forest plot of subgroup analyses for gait speed.

Subgroup analyses identified several important factors that may influence the intervention effects. The relevant results are shown in Table 2, and the corresponding forest plots are presented in Appendix 4. For knee extension strength (Figure 5), there was a statistically significant subgroup difference across types of sarcopenia (χ^2^ = 13.76, *p* = 0.006). Patients with sarcopenic obesity exhibited a greater improvement in knee extension strength (SMD = 1.81, 95% CI: 1.11 to 2.51) compared to those with sarcopenia alone (SMD = 0.72, 95% CI: 0.38 to 1.06). For gait speed (Figure 6), statistically significant subgroup differences were observed for types of sarcopenia (χ^2^ = 6.52, *p* = 0.01). Specifically, resistance training significantly improved gait speed in women with sarcopenia alone (SMD = 0.61, 95% CI: 0.30 to 0.92), whereas no significant improvement was observed in those with sarcopenic obesity (SMD = 0.02, 95% CI: −0.30 to 0.35). Additionally, the types of resistance training (χ^2^ = 16.54, *p* = 0.06) showed statistically significant subgroup differences. Compared with constant resistance training (SMD = −0.06, 95% CI: −0.54 to 0.43) and variable resistance training (SMD = 0.38, 95% CI: −0.30 to 1.07), mixed resistance training (SMD = 0.61, 95% CI: 0.34 to 0.89) demonstrated a greater advantage in improving gait speed (Table 3).

**Table 3:** Summary of subgroup analysis results.

Outcome	Number of participants	Heterogeneity		Meta analysis		Subgroup differences
		*p*-value	*I^2^*	Effect estimate (95%CI)	*P*-value	
Meta-analysis results by types of sarcopenia						
Handgrip strength	327	0.07*	46.60%	0.43(0.11,0.74)	0.007*	0.32
Sarcopenic	251	0.03*	59.30%	0.54(0.10,0.97)		
Sarcopenic obesity	76	0.58	0.00%	0.22(−0.23,0.67)		
Knee extension strength	342	0.002*	72.10%	0.85(0.37,1.33)	<0.001*	<0.001*
Sarcopenic	268	0.75	0.00%	0.52(0.27,0.76)		
Sarcopenic obesity	74	0.21	36.40%	1.81(1.11,2.51)		
Gait speed	425	0.04*	51.60%	0.37(0.09,0.64)	0.009*	0.011*
Sarcopenic	275	0.09*	50.30%	0.61(0.30,0.92)		
Sarcopenic obesity	150	0.86	0.00%	0.02(−0.30,0.35)’		
Timed Up and Go test	222	0.24	26.70%	−0.68(−0.95,-0.41)	<0.001*	0.37
Sarcopenic	100	0.067	70.40%	−1.03(−1.98,-0.08)		
Sarcopenic obesity	122	0.52	0.00%	−0.57(−0.93,-0.21)		
Skeletal muscle index	240	0.59	0.00%	0.13(−0.13,0.38)	0.34	0.39
Sarcopenic	111	0.61	0.00%	0.04(−0.34,0.41)		
Sarcopenic obesity	129	0.31	13.60%	0.24(−0.20,0.67)		
Meta-analysis results by setting						
Handgrip strength	327	0.07*	46.60%	0.43(0.11,0.74)	0.007*	0.69
Institution	146	0.03*	72.40%	0.57(−0.21,1.34)		
Community	181	0.21	31.50%	0.39(0.01,0.77)		
Knee extension strength	342	0.002*	72.10%	0.85(0.37,1.33)	<0.001*	0.3
Institution	122	<0.001*	90.00%	1.38(−0.06,2.81)		
Community	220	0.21	31.70%	0.59(0.26,0.92)		
Gait speed	425	0.04*	51.60%	0.37(0.09,0.64)	0.009*	0.37
Institution	192	0.03*	66.90%	0.56(−0.02,1.14)		
Community	233	0.15	40.10%	0.25(−0.10,0.60)		
Skeletal muscle index	240	0.59	0.00%	0.13(−0.13,0.38)	0.34	0.61
Institution	118	0.35	8.00%	0.19(−0.21,0.60)		
Community	122	0.69	0.00%	0.06(−0.30,0.41)		
Meta-analysis results by types of resistance						
Handgrip strength	263	0.08*	46.40%	0.51(0.15,0.86)	0.005*	0.83
Variable resistance	100	0.04*	69.00%	0.58(−0.23,1.40)		
Constant resistance	163	0.19	38.60%	0.48(0.05,0.92)		
Knee extension strength	296	0.3	17.00%	0.59(0.36,0.83)	<0.001*	0.28
Mixed resistance	213	0.39	0.00%	0.50(0.23,0.78)		
Constant resistance	83	0.26	26.60%	0.84(0.29,1.40)		
Gait speed	425	0.04*	51.60%	0.37(0.09,0.64)	0.009*	0.06*
Variable resistance	146	0.36	68.60%	0.38(−0.30,1.07)		
Constant resistance	66	0.64	0.00%	−0.06(−0.54,0.43)		
Mixed resistance	213	0.78	0.00%	0.61(0.34,0.89)		
Skeletal muscle index	240	0.59	0.00%	0.13(−0.13,0.38)	0.34	0.49
Variable resistance	111	0.61	0.00%	0.04(−0.34,0.41)		
Constant resistance	129	0.31	13.60%	0.24(−0.20,0.67)		

Statistical significance for subgroup interactions was set at *p* < 0.1 due to limited statistical power. *p*-values for heterogeneity were interpreted using a threshold of *P* < 0.1, whereas *P*-values for pooled effect estimates were considered statistically significant at *p* < 0.05. * Indicates statistical significance.

### 3.6 Sensitivity analysis

Sensitivity analyses were conducted to assess the influence of individual studies on pooled estimates and heterogeneity. Meta-analysis results under both fixed-effect and random-effects models were reported. The direction of effect estimates remained consistent across models, with small differences in SMD, suggesting low sensitivity to model assumptions and strong robustness (Figures 3, 4).

**Figure 7:**
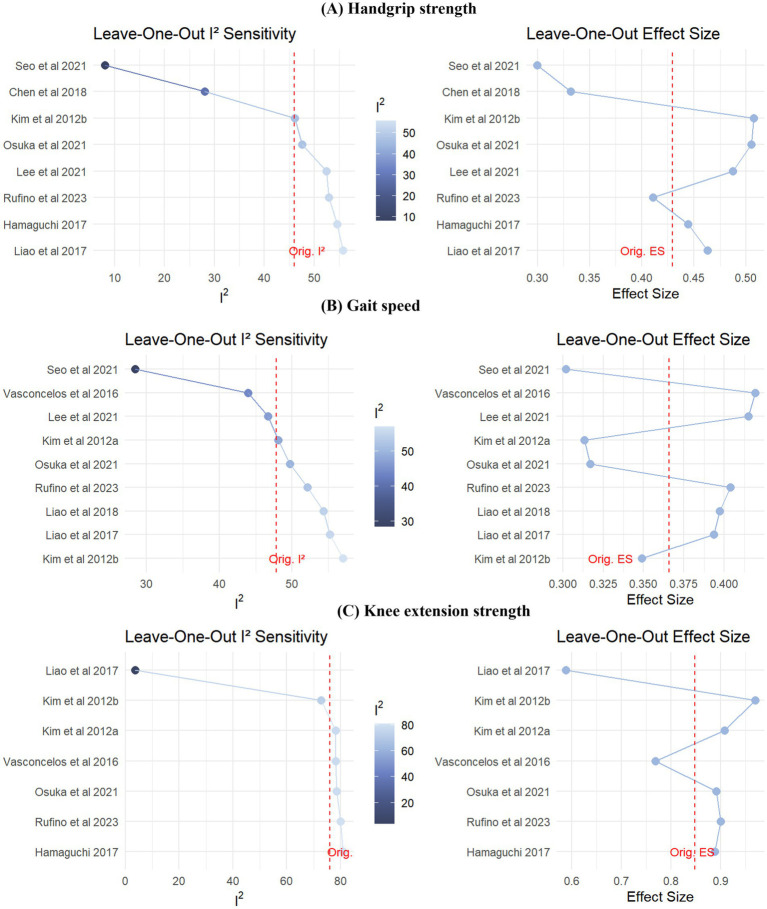
Leave-one-out sensitivity analyses for **(A)** Handgrip strength, **(B)** Gait speed, and **(C)** knee extension strength: impact on heterogeneity (*I*^2^) and effect size.

We further performed a leave-one-out sensitivity analysis to examine each study’s influence. Results indicated that Seo et al. (39) mainly contributed to heterogeneity in handgrip strength and gait speed outcomes. Liao et al. (40) accounted for most heterogeneity in knee extension strength. Excluding these studies substantially reduced heterogeneity in the respective outcomes. Although effect sizes fluctuated slightly, the results’ overall direction and statistical significance remained unchanged, further supporting the robustness and reliability of our findings (Figure 7 and Appendix 5).

### 3.7 Publication bias

To assess the potential impact of publication bias, Egger’s regression asymmetry test was conducted for both primary and secondary outcomes (Appendix 3). The results indicated possible publication bias for handgrip strength (*p* = 0.0733); however, the trim-and-fill method did not impute any missing studies, suggesting that the original findings are robust and that potential publication bias is unlikely to have materially affected the conclusions. No significant asymmetry was detected for other outcomes, including knee extension strength (*p* = 0.2331), gait speed (*p* = 0.7470), TUG (*p* = 0.4044), CST (*p* = 0.4660), and SMI (*p* = 0.7090).

### 3.8 Adverse events

Three studies reported adverse events related to resistance training, all of which were mild and did not lead to study dropout or serious health problems. Liao et al. (40) reported mild knee or back pain in two participants, which resolved after adjusting exercise intensity and technique. Rufino et al. (41) noted muscle discomfort in three participants during early high-intensity sessions, which resolved after lowering training loads. Vasconcelos et al. (42) recorded four cases of back pain, soreness, or cramping, primarily in early sessions; symptoms were mitigated by posture correction and gradual adaptation. All reported adverse events were transient, mild, and reversible, and no serious adverse events or dropouts due to intervention were reported, indicating good safety and tolerability of resistance training in this population.

## 4 Discussion

### 4.1 Summary of findings

This systematic review included 12 RCTs involving a total of 518 older women with a confirmed diagnosis of sarcopenia. The meta-analysis demonstrated that resistance training significantly improves muscle strength and physical function in this population. However, no statistically significant improvements were observed in muscle mass indicators, such as the SMI. Subgroup analyses suggested that the effectiveness of the intervention may be influenced by the sarcopenia subtype and the types of resistance training.

### 4.2 Comparison with other studies

Previous meta-analyses have evaluated the effects of exercise training on healthy middle-aged women. Park et al. (22) found that exercise slightly improved body composition (ES = 0.147) and had a moderate effect on enhancing physical activity capacity (ES = 0.510), with resistance training showing superior outcomes compared to aerobic training in overall sarcopenia-related measures (ES = 0.354 vs. 0.096). Tan et al. (21) further reported that exercise significantly improved muscle strength and mass, including lean mass (ES = 0.232), handgrip strength (ES = 0.901), and knee extension strength (ES = 0.698), with resistance training showing the most prominent effect on lean mass (ES = 0.316). Consistent with those findings, our study confirmed the positive effects of resistance training on muscle strength. Notably, in some included trials, the between-group difference appeared to be driven mainly by prevention of deterioration in the control condition rather than large absolute gains in the RT group (43). This suggests that, in more vulnerable subgroups or those at higher risk of decline, preserving strength may itself constitute a clinically relevant outcome. However, no significant improvement in muscle mass was observed in older women with sarcopenia. Several physiological factors specific to this population may explain the discrepancy between strength gains and muscle mass stability. First, strength improvements in older adults are often primarily driven by neural adaptations, such as improved motor unit recruitment, rather than structural hypertrophy. This is particularly relevant given that neural adaptations typically precede muscle growth in the early phases of training (44). Second, physiological changes associated with menopause create a challenging environment for muscle hypertrophy. The drastic decline in estrogen levels impairs satellite cell function and exacerbates anabolic resistance, a condition characterized by a blunted muscle protein synthesis response to mechanical loading and amino acid intake. Consequently, standard training volumes that induce hypertrophy in other populations might be insufficient to overcome this diminished sensitivity in older women (44, 45). To further enhance training efficacy, recent studies propose combining resistance training with nutritional strategies, particularly protein supplementation, to amplify neuromuscular adaptations (46–48). For instance, Cuyul-Vásquez et al. (46) found that protein supplementation combined with resistance training was more effective than resistance training alone in improving appendicular skeletal muscle index and handgrip strength. Future studies should explore the interaction between protein type, dosage, and training parameters to optimize comprehensive intervention strategies.

Despite the challenges in achieving hypertrophy, prioritizing physical function remains the primary clinical goal, as evidence suggests that declines in muscle function, rather than muscle mass per se, are the primary drivers of disability in older adults (49). Therefore, resistance training programs for this population should prioritize improvements in strength and function over muscle hypertrophy alone. Some evidence suggests that variable resistance training may be more effective than constant resistance training in improving physical function among older adults (50). However, subgroup analysis in the present study found that neither of these two training modalities significantly improved gait speed. This lack of significance may be attributed to the multifactorial nature of gait speed in older adults. Walking performance is often constrained by non-muscular factors, including chronic conditions such as vestibular dysfunction, visual impairment, and the fear of falling (51, 52). These comorbidities are common in geriatric populations and can act as a functional ceiling that limits the transfer of strength gains to walking speed when the training stimulus is monotonic. In contrast, Mixed resistance training demonstrated a clear advantage in this outcome. This is likely because mixed training combines the mechanical benefits of different loading profiles. It provides both the high mechanical tension of constant resistance and the peak contraction velocity of variable resistance. This complementary adaptation targets the full force-velocity spectrum and generates a robust neuromuscular stimulus. Such a comprehensive stimulus may be necessary to elicit functional improvements despite the presence of sensory or psychological barriers (53, 54).

It is worth noting that constant resistance training not only failed to improve gait speed but may even lead to further deterioration. The effect of resistance training also appeared to be limited among older women with sarcopenic obesity. Significant improvement was observed only in knee extension strength, while no significant effects were detected in other key outcomes. Previous studies have shown that individuals with sarcopenic obesity are at higher risk of metabolic disorders compared with those with sarcopenia or obesity alone. They also tend to have a higher prevalence of cardiovascular disease and all-cause mortality, with more pronounced physical decline, often characterized by reduced gait speed and impaired balance (55–57). This functional impairment may be associated with abnormal fat deposition within and around muscle tissue as aging and obesity progress, which can disrupt muscle structure and metabolic function, reduce muscle mass, and weaken strength output relative to muscle quantity (58, 59). This phenomenon, known as myosteatosis, has been widely recognized as a key physiological mechanism contributing to physical functional limitations in older adults (55, 60). Therefore, for individuals with sarcopenic obesity, relying solely on resistance training may be insufficient to achieve comprehensive improvements in physical function. Future interventions could consider incorporating balance training into resistance programs, using more diverse resistance patterns, or combining resistance training with nutritional support to enhance the overall effectiveness and adaptability of interventions (61, 62).

Notably, two included studies reported mild adverse symptoms at the beginning of the intervention, which resolved after adjusting training intensity and duration. Therefore, resistance training programs for older adults should follow individualization, periodization, and progressive overload principles, with careful attention to load tolerance and recovery (63). Most studies in older adults have used training intensities ranging from 30 to 90% of one-repetition maximum (1RM) (64). While higher intensities (70–85% 1RM) are often necessary for inducing muscle hypertrophy and neuromuscular adaptations, such protocols may be poorly tolerated by frail or functionally impaired individuals and could increase the risk of adverse events (65). High-load strategies may also be less feasible and cost-effective in this population. Rather than focusing solely on training intensity, programs emphasizing diverse exercise modalities and alignment with individual functional goals may offer more comprehensive health benefits for older adults with sarcopenia.

### 4.3 Strengths and limitations

This study represents the first systematic review and meta-analysis specifically focused on older women with sarcopenia. The overall certainty of the evidence was high, with all outcomes rated as moderate to high quality. Egger’s test indicated potential publication bias only for handgrip strength, while the trim-and-fill method did not identify any missing studies, suggesting that the impact of bias on the pooled effect estimates is likely limited. Robustness of the findings was further supported by two forms of sensitivity analysis. Moreover, no serious adverse events were reported in any of the included trials during the intervention period, reinforcing the safety and tolerability of resistance training in this population. Taken together, this study provides reliable and practically relevant evidence to guide exercise-based interventions for older women with sarcopenia.

However, several limitations should be acknowledged. First, the included studies used heterogeneous diagnostic criteria for sarcopenia, potentially contributing to clinical heterogeneity. As Smith et al. (66) argued, differing diagnostic definitions should not preclude evaluation of intervention effectiveness; instead, emphasis should be placed on intervention design, delivery, and adherence. Second, follow-up durations were generally short, limiting conclusions about long-term efficacy. Finally, the number of high-quality RCTs focusing on this specific population remains limited, and small sample sizes may affect the stability and generalizability of effect estimates. Future research should include larger, longer-term trials to confirm these findings.

## 5 Conclusion

This systematic review and meta-analysis indicates that resistance training is an effective intervention for improving muscle strength and physical function in older women with sarcopenia. Among different modalities, combined resistance training may offer greater benefits in enhancing dynamic physical performance. For individuals with sarcopenic obesity, traditional resistance training alone may be insufficient to achieve optimal outcomes. Future interventions are encouraged to integrate multicomponent exercise programs, such as balance and aerobic training, together with nutritional support in order to enhance overall effectiveness and improve adaptability in these populations.

## Footnotes

1
www.gradepro.org
