# Supplementary material for: Effects of resistance training on muscle mass, strength, and physical function in older women with sarcopenia: a systematic review and meta-analysis
Source: Front Public Health. 2026 Jan 26;13:1735899. doi: 10.3389/fpubh.2025.1735899 (PMC12883749; doi:10.3389/fpubh.2025.1735899)

Supplementary Appendix

**Effects of Resistance Training on Muscle Mass, Strength, and Physical Function in Older Women With Sarcopenia: A Systematic Review and Meta-Analysis**

**Table of contents**

[Appendix 1: Search strategy 2](#_Toc21294)

[Appendix 2: Risk of bias of randomized clinical trials 6](#_Toc25229)

[Appendix 3: Funnel Plots with Egger’s Test for Publication Bias 7](#_Toc3676)

[Appendix 4: Subgroup Analysis 10](#_Toc14935)

[Appendix 5: Sensitivity analysis 17](#_Toc12447)

# Appendix 1: Search strategy

**Table S1.1** Search strategy of Pubmed

| **#** | **Searches** |
| --- | --- |
| 1 | (((((((((((((((((((((((Training, Resistance[Title/Abstract]) OR (Strength Training[Title/Abstract])) OR (Training, Strength[Title/Abstract])) OR (Weight-Lifting Strengthening Program[Title/Abstract])) OR (Strengthening Programs, Weight-Lifting[Title/Abstract])) OR (Strengthening Program, Weight-Lifting[Title/Abstract])) OR (Weight Lifting Strengthening Program[Title/Abstract])) OR (Weight-Lifting Strengthening Programs[Title/Abstract])) OR (Weight-Lifting Exercise Program[Title/Abstract])) OR (Exercise Programs, Weight-Lifting[Title/Abstract])) OR (Exercise Program, Weight-Lifting[Title/Abstract])) OR (Weight Lifting Exercise Program[Title/Abstract])) OR (Weight-Lifting Exercise Programs[Title/Abstract])) OR (Weight-Bearing Strengthening Program[Title/Abstract])) OR (Strengthening Programs, Weight-Bearing[Title/Abstract])) OR (Strengthening Program, Weight-Bearing[Title/Abstract])) OR (Weight Bearing Strengthening Program[Title/Abstract])) OR (Weight-Bearing Strengthening Programs[Title/Abstract])) OR (Weight-Bearing Exercise Program[Title/Abstract])) OR (Exercise Programs, Weight-Bearing[Title/Abstract])) OR (Exercise Program, Weight-Bearing[Title/Abstract])) OR (Weight Bearing Exercise Program[Title/Abstract])) OR (Weight-Bearing Exercise Programs[Title/Abstract])) OR (Resistance training[MeSH Terms]) |
| 2 | (((Sarcopenia[MeSH Terms]) OR (sarcopenia[Title/Abstract])) OR (Muscle loss[Title/Abstract])) OR (Sarcopenias[Title/Abstract]) |
| 3 | (Women[MeSH Terms]) OR (Female[MeSH Terms]) |
| 4 | ((Older[Title/Abstract]) OR (Aged[MeSH Terms])) OR (Elderly[Title/Abstrac]) |
| 5 | #1 AND #2 AND #3 AND #4 |

**Table S1.2** Search strategy of Web of Science

| **#** | **Searches** |
| --- | --- |
| 1 | TS=("Sarcopenia" OR "Sarcopenias" OR "Muscle loss") |
| 2 | TS=("Resistance training" OR "Training, Resistance" OR "Strength Training" OR "Training, Strength" OR "Weight-Lifting Strengthening Program" OR "Strengthening Programs, Weight-Lifting" OR "Strengthening Program, Weight-Lifting" OR "Weight Lifting Strengthening Program" OR "Weight-Lifting Strengthening Programs" OR "Weight-Lifting Exercise Program" OR "Exercise Programs, Weight-Lifting" OR "Exercise Program, Weight-Lifting" OR "Weight Lifting Exercise Program" OR "Weight-Lifting Exercise Programs" OR "Weight-Bearing Strengthening Program" OR "Strengthening Programs, Weight-Bearing" OR "Strengthening Program, Weight-Bearing" OR "Weight Bearing Strengthening Program" OR "Weight-Bearing Strengthening Programs" OR "Weight-Bearing Exercise Program" OR "Exercise Programs, Weight-Bearing" OR "Exercise Program, Weight-Bearing" OR "Weight Bearing Exercise Program" OR "Weight-Bearing Exercise Programs") |
| 3 | TS=("Women" OR "Female") |
| 4 | TS=("Older" OR "Elderly" OR "Aged") |
| 5 | #1 AND #2 AND #3 AND #4 |

**Table S1.3** Search strategy of Cochrane Central Register of Controlled Trials

| **#** | **Searches** |
| --- | --- |
| 1 | MeSH descriptor: [Sarcopenia] explode all trees |
| 2 | MeSH descriptor: [Resistance Training] explode all trees |
| 3 | MeSH descriptor: [Female] explode all trees |
| 4 | MeSH descriptor: [Women] explode all trees |
| 5 | MeSH descriptor: [Aged] explode all trees |
| 6 | #3 OR #4 |
| 27 | #1 AND #2 AND #5 AND #6 |

**Table 1.4** Search strategy of Embase

| **#** | **Searches** |
| --- | --- |
| 1 | 'Sarcopenia'/exp OR 'sarcopenia':ab,ti OR 'Muscle loss':ab,ti OR 'sarcopenias':ab,ti |
| 2 | 'Resistance training'/exp OR 'Resistance training':ab,ti OR 'Training, Resistance':ab,ti OR 'Strength Training':ab,ti OR 'Training, Strength':ab,ti OR 'Weight-Lifting Strengthening Program':ab,ti OR 'Strengthening Programs, Weight-Lifting':ab,ti OR 'Strengthening Program, Weight-Lifting':ab,ti OR 'Weight Lifting Strengthening Program':ab,ti OR 'Weight-Lifting Strengthening Programs':ab,ti OR 'Weight-Lifting Exercise Program':ab,ti OR 'Exercise Programs, Weight-Lifting':ab,ti OR 'Exercise Program, Weight-Lifting':ab,ti OR 'Weight Lifting Exercise Program':ab,ti OR 'Weight-Lifting Exercise Programs':ab,ti OR 'Weight-Bearing Strengthening Program':ab,ti OR 'Strengthening Programs, Weight-Bearing':ab,ti OR 'Strengthening Program, Weight-Bearing':ab,ti OR 'Weight Bearing Strengthening Program':ab,ti OR 'Weight-Bearing Strengthening Programs':ab,ti OR 'Weight-Bearing Exercise Program':ab,ti OR 'Exercise Programs, Weight-Bearing':ab,ti OR 'Exercise Program, Weight-Bearing':ab,ti OR 'Weight Bearing Exercise Program':ab,ti OR 'Weight-Bearing Exercise Programs':ab,ti |
| 3 | 'Female'/exp OR 'Women'/exp |
| 4 | 'Aged'/exp OR 'Older':ab,ti OR 'Elderly':ab,ti |
| 4 | #1 AND #2 AND #3 AND #4 |

# Appendix 2: Risk of bias of randomized clinical trials

**Table S2.1:** Study level risk of bias assessment using Cochrane risk of bias tool 2.0 for assessing risk of bias of randomized clinical trials.

| Unique ID | Randomization process | Deviations from intended interventions | Missing outcome data | Measurement of the outcome | Selection of the reported result | Overall Bias |
| --- | --- | --- | --- | --- | --- | --- |
| Huang et al2017 | Low | Low | Low | Low | Low | Low |
| Liao et al 2017 | Some concerns | Low | Low | Low | Some concerns | Some concerns |
| Lee et al2021 | Low | Low | Low | Low | Low | Low |
| Osuka et al2021 | Low | Low | Low | Low | Low | Low |
| Seo et al2021 | Some concerns | Low | Low | Low | Some concerns | Some concerns |
| Vasconcelos et al2016 | Low | Low | Low | Low | Low | Low |
| Rufino et al2023 | Low | Low | Low | Low | Low | Low |
| Hamaguchi et al2017 | Some concerns | Some concerns | Low | Low | Low | Some concerns |
| Chen et al 2018 | Some concerns | Low | Low | Low | Some concerns | Some concerns |
| Kim et al2012a | Some concerns | Low | Low | Low | Some concerns | Some concerns |
| Kim et al2012b | Low | Low | Low | Low | Low | Low |
| Liao et al2018 | Low | Low | Low | Low | Low | Low |

# Appendix 3: Funnel Plots with Egger’s Test for Publication Bias

**Figure S3.1:** Funnel plot of Handgrip strength


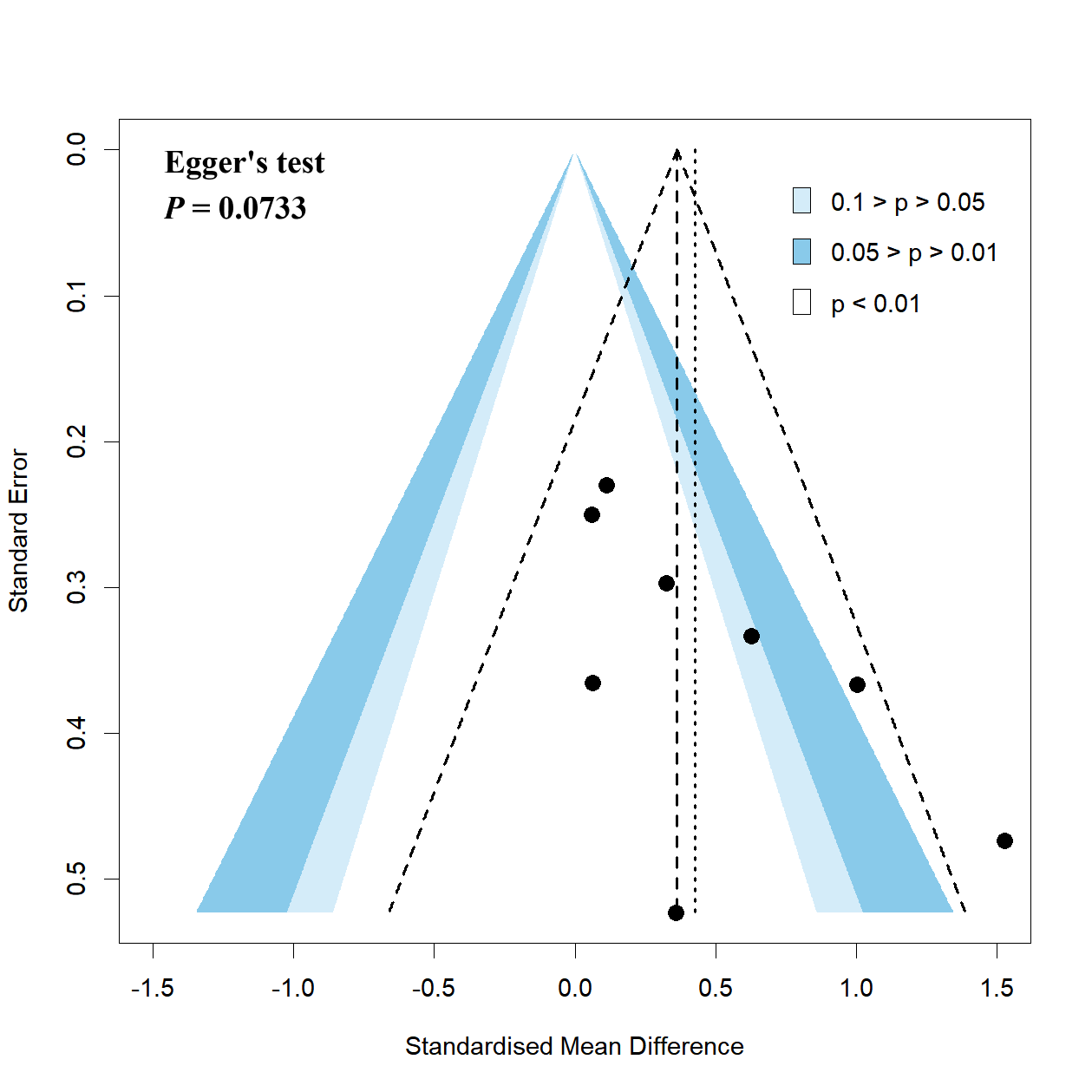


**Figure S3.2:** Funnel plot of Knee extension strength


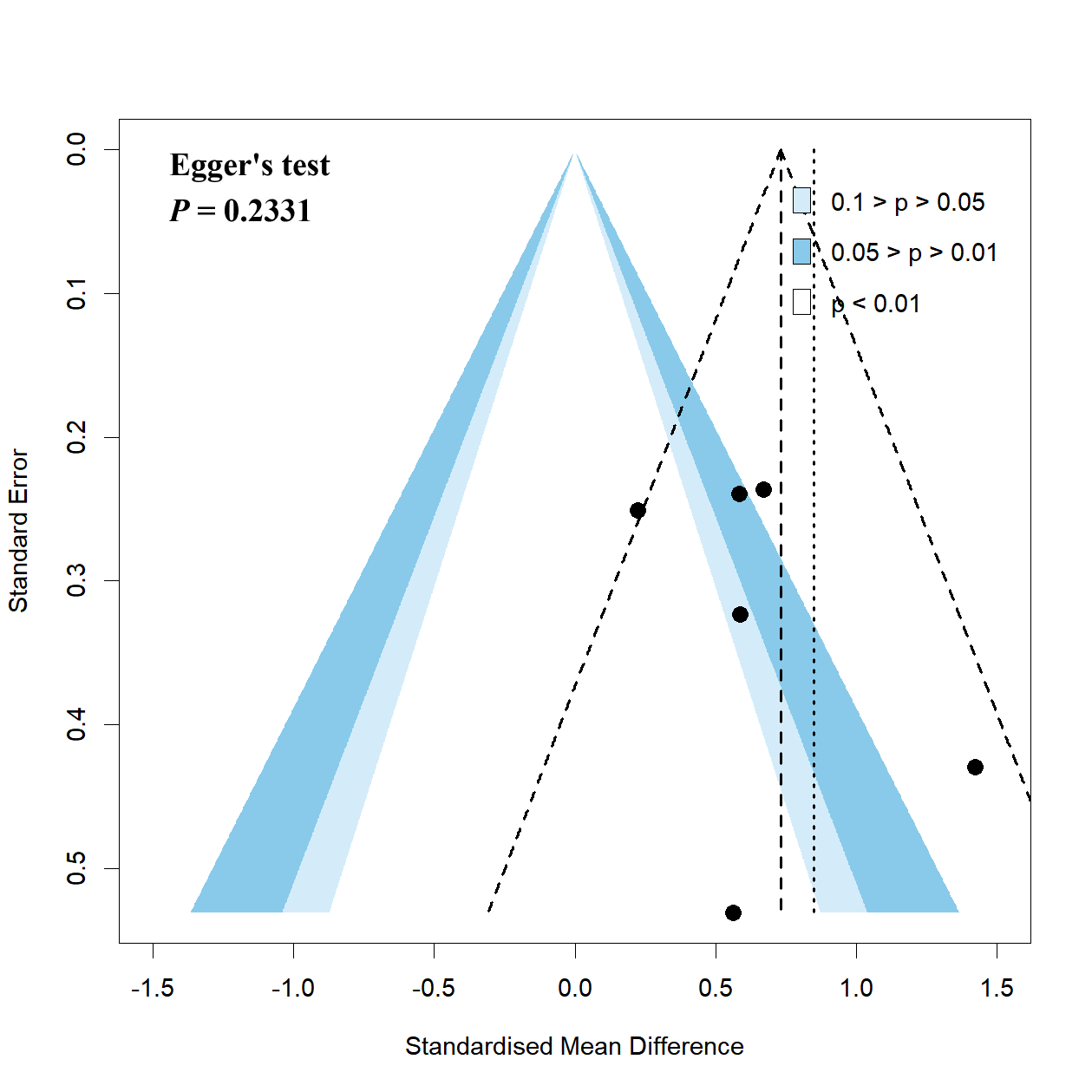


**Figure S3.3:** Funnel plot of Gait speed


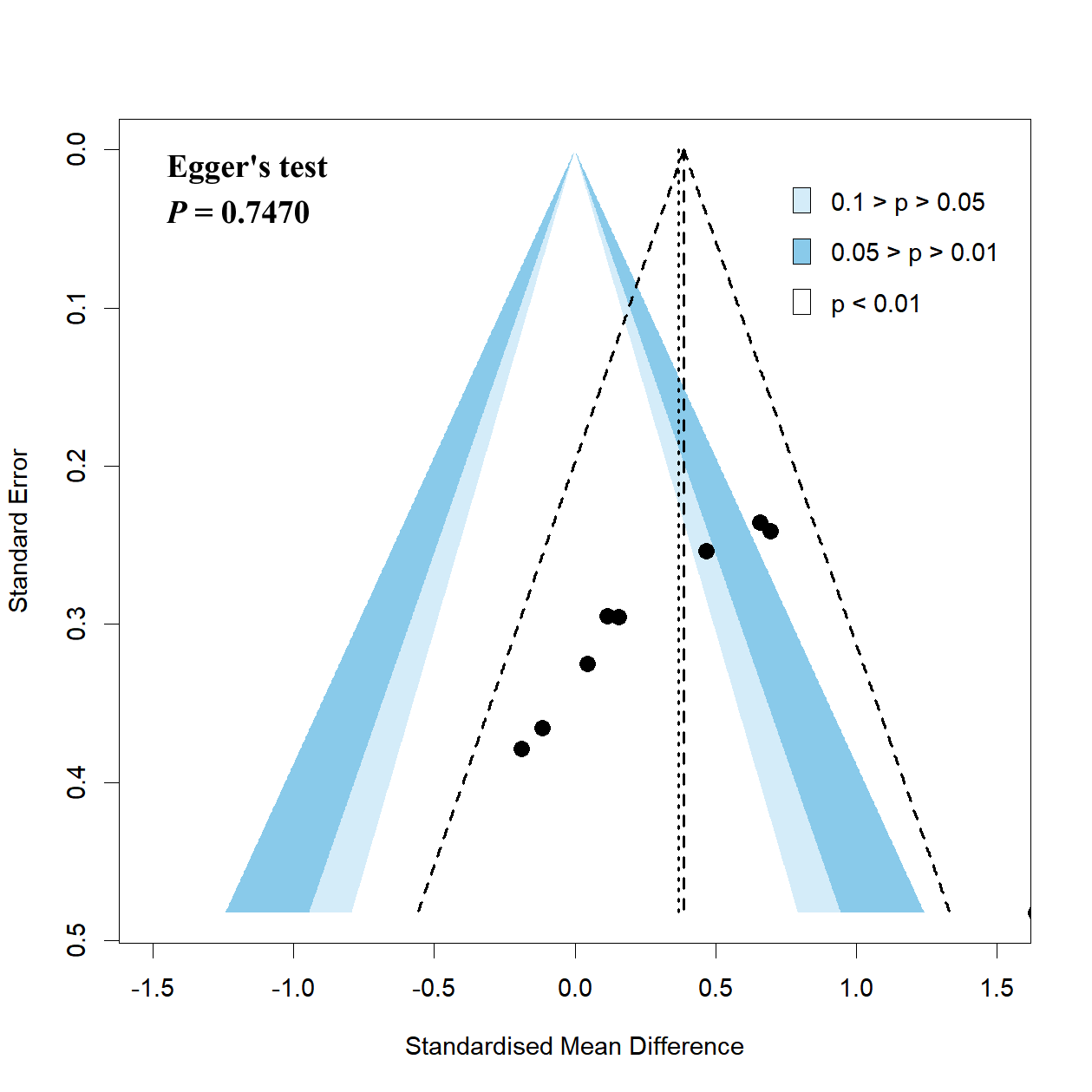


**Figure S3.4:** Funnel plot of TUG


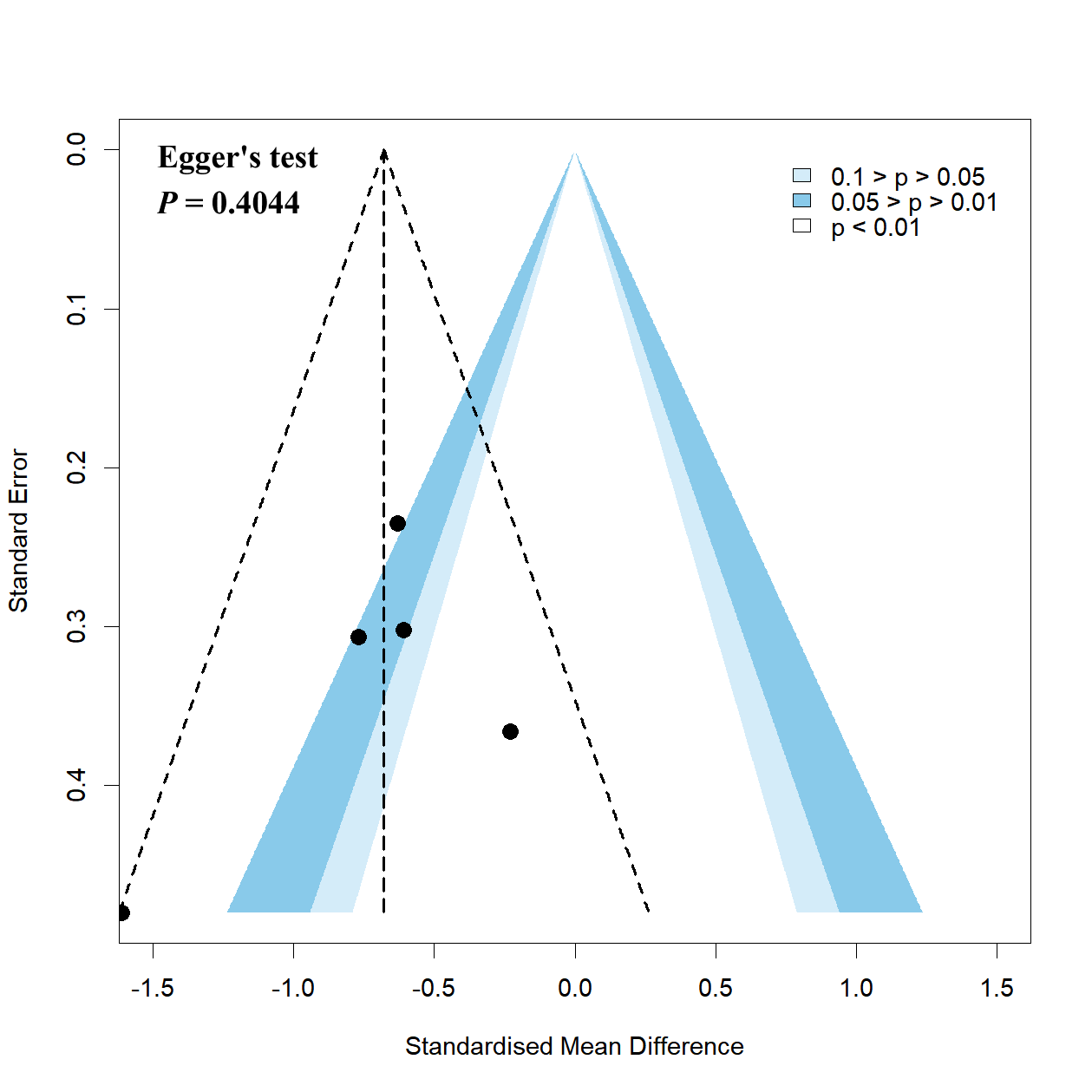


**Figure S3.5:** Funnel plot of CST


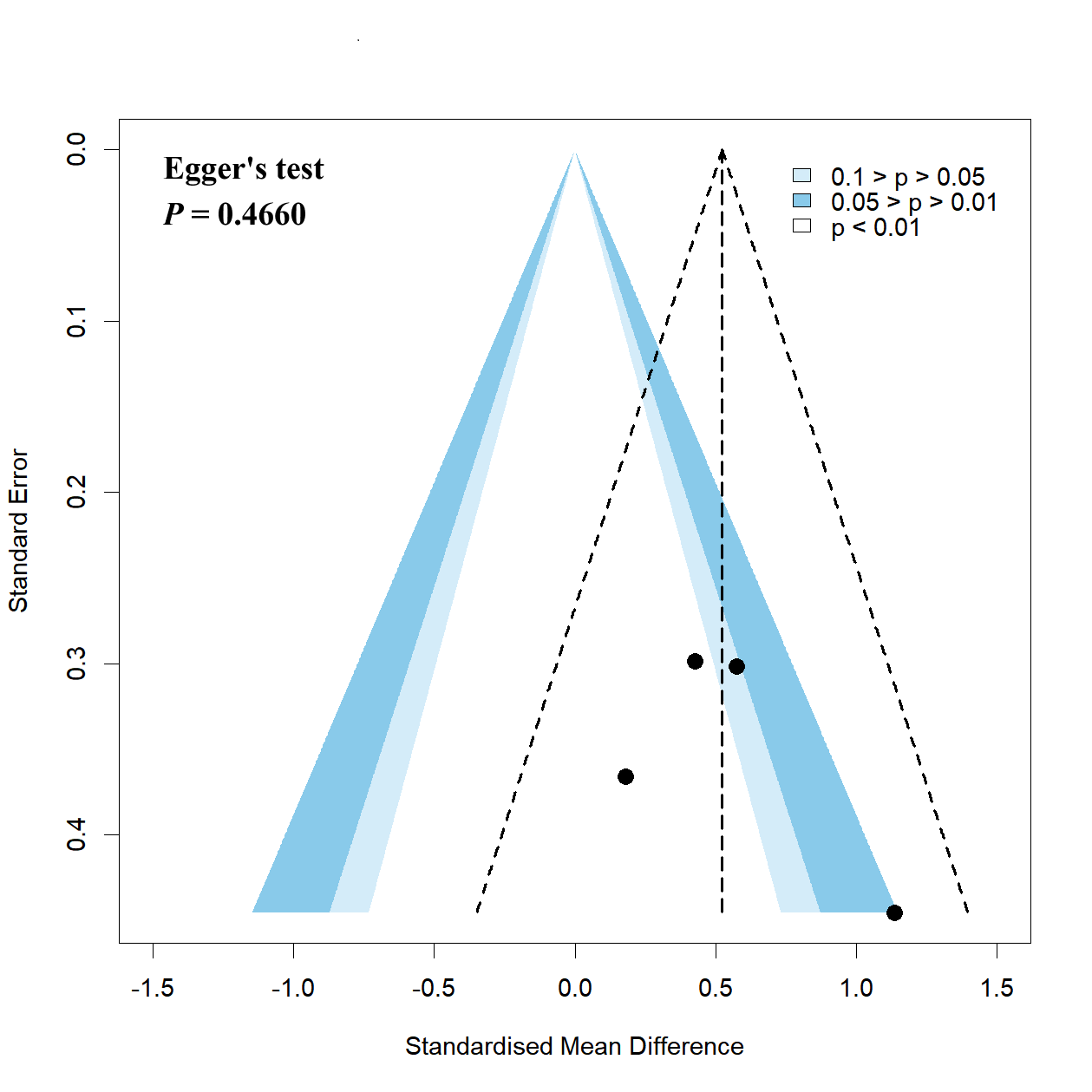


**Figure S3.6:** Funnel plot of SMI


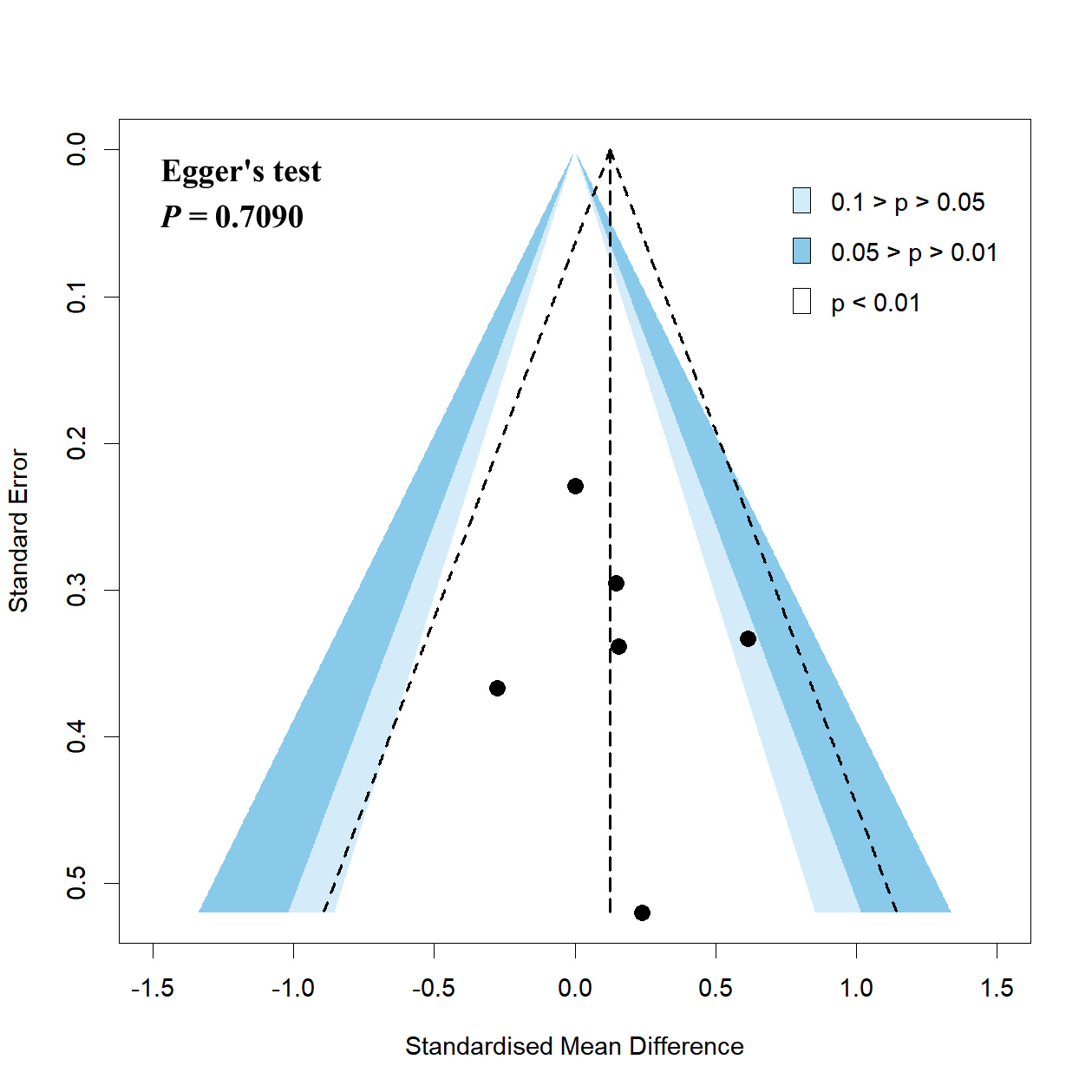


# Appendix 4: Subgroup Analysis

**Appendix 4.1 Subgroup Analysis by Setting**

**Figure S4.1.1:** Funnel plot of Handgrip strength


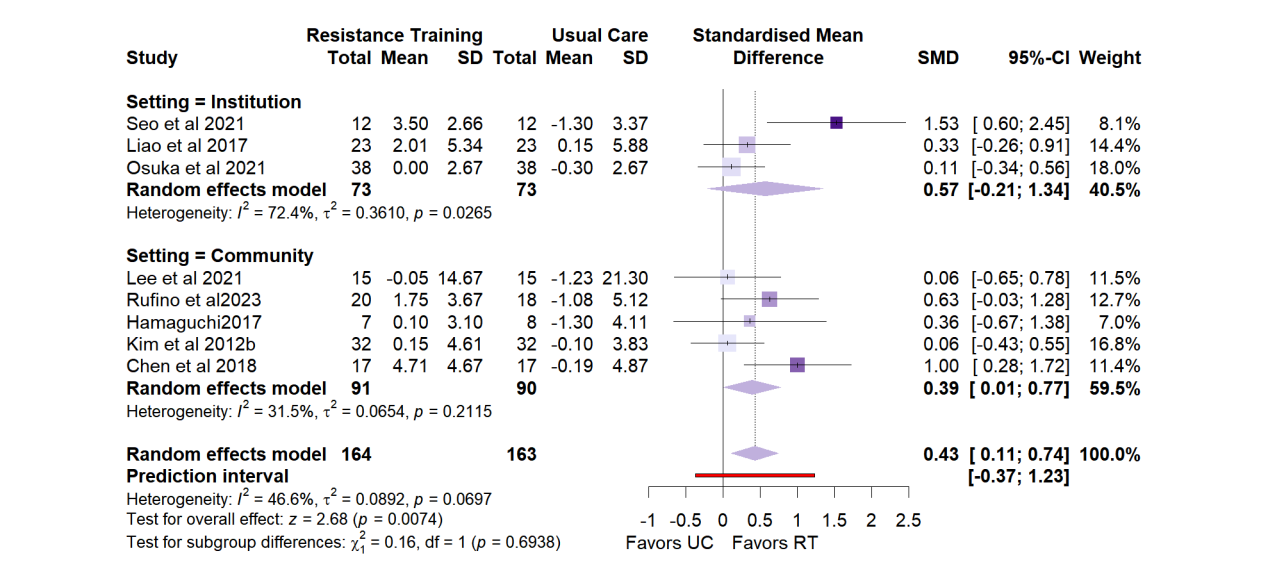


**Figure S4.1.2:** Funnel plot of Knee extension strength


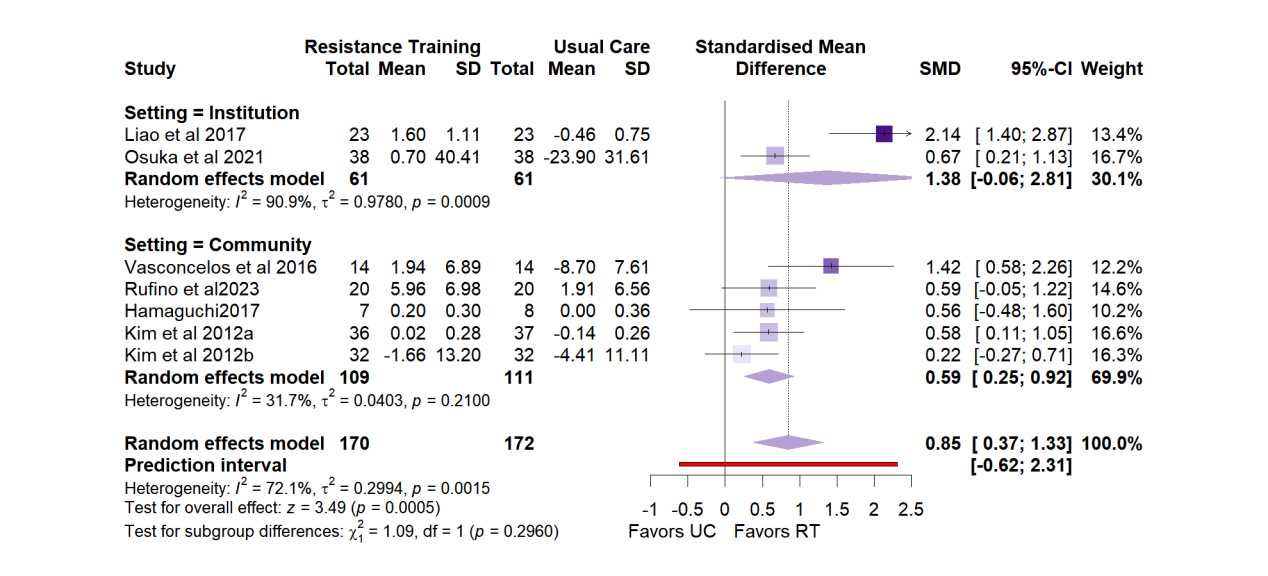


**Figure S4.1.3:** Funnel plot of Gait speed


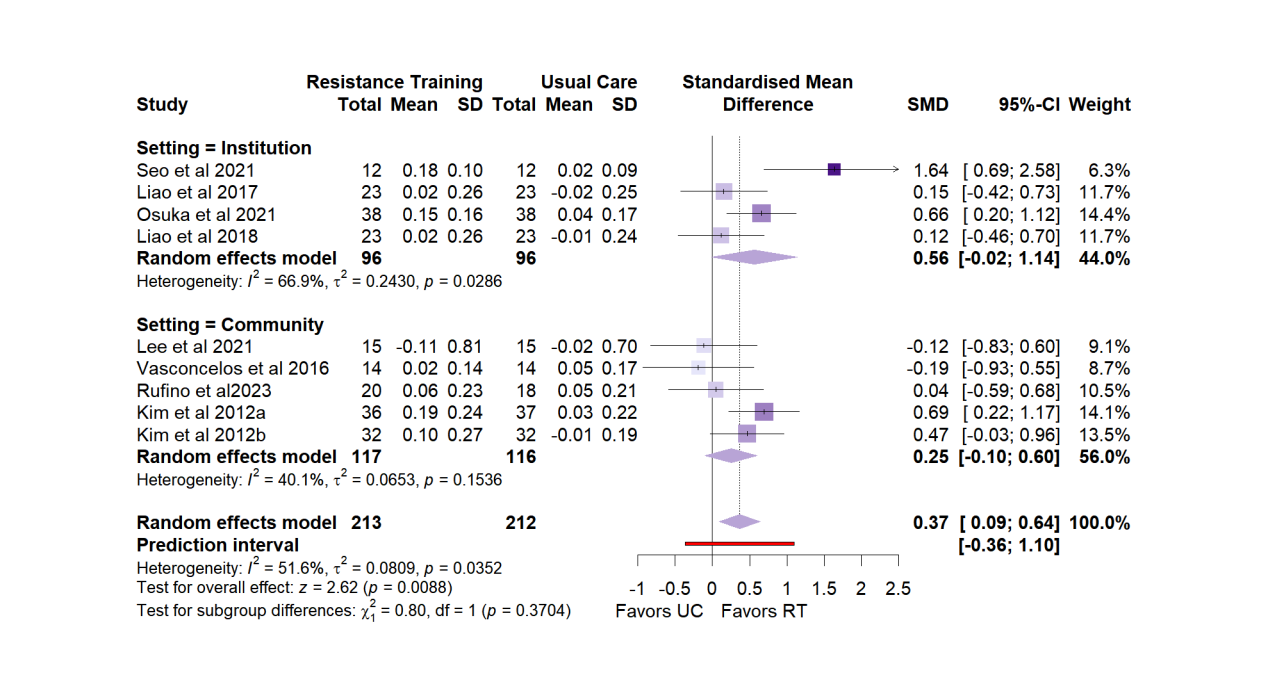


**Figure S4.1.4:** Funnel plot of SMI


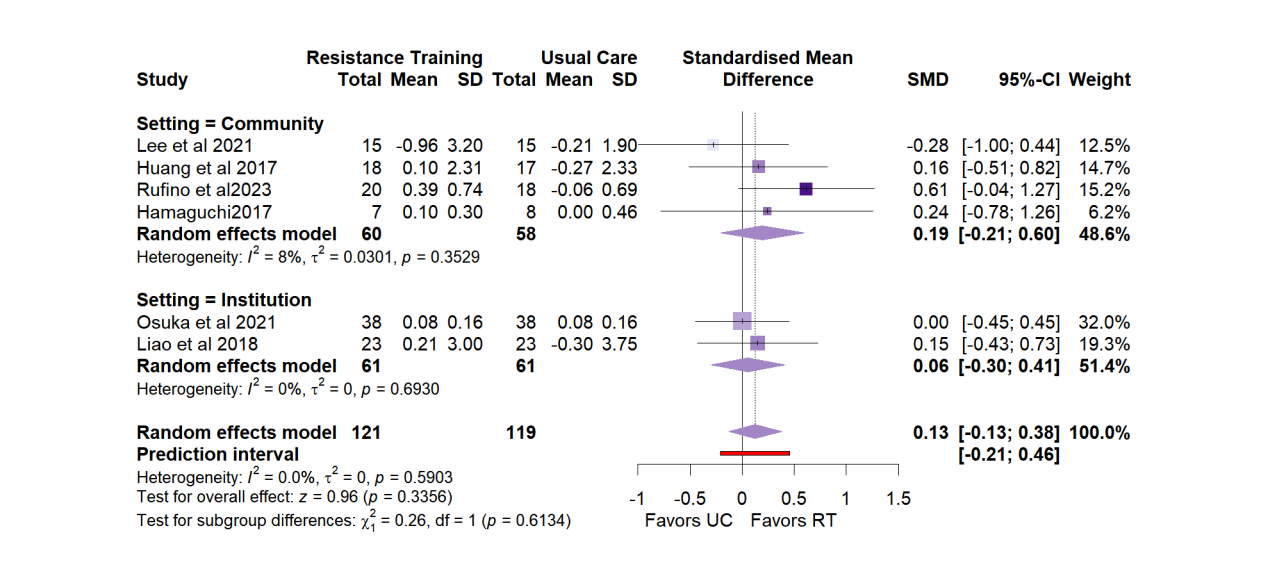


**Appendix 4.2 Subgroup Analysis by type of sarcopenia**

**Figure S4.2.1:** Funnel plot of Handgrip strength


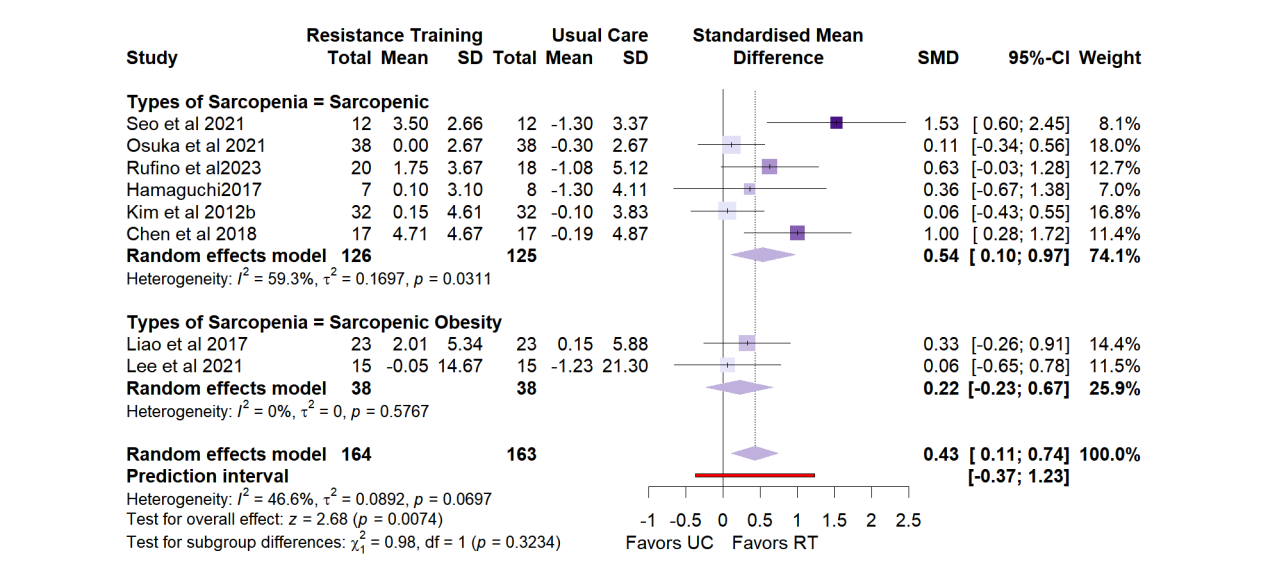


**Figure S4.2.2:** Funnel plot of Knee extension strength


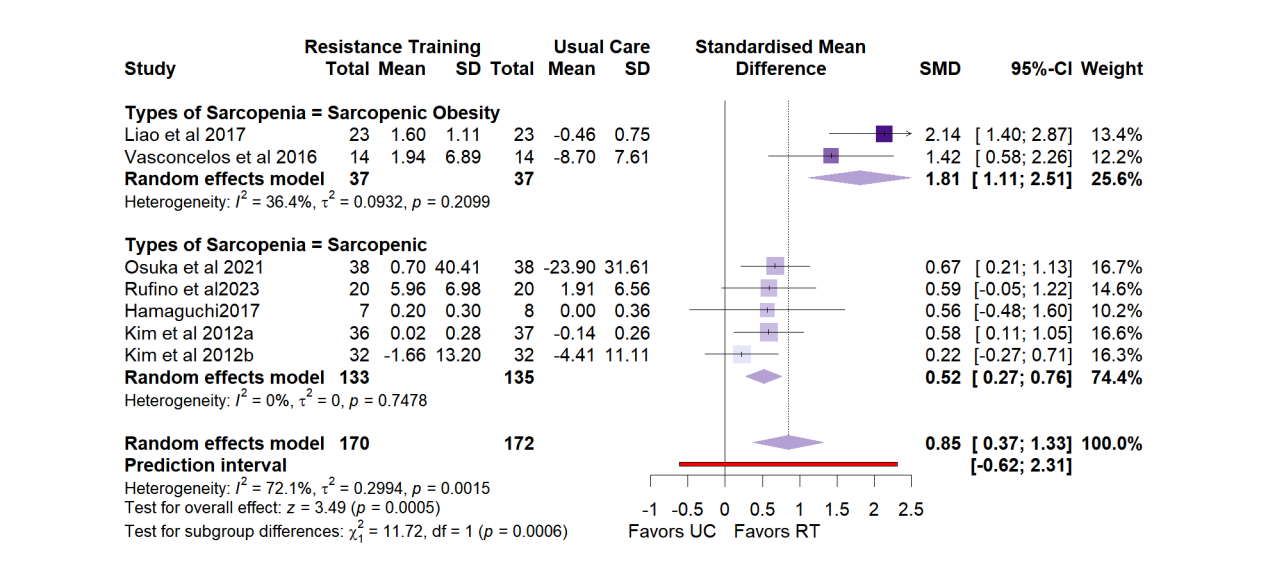


**Figure S4.2.3:** Funnel plot of Gait speed


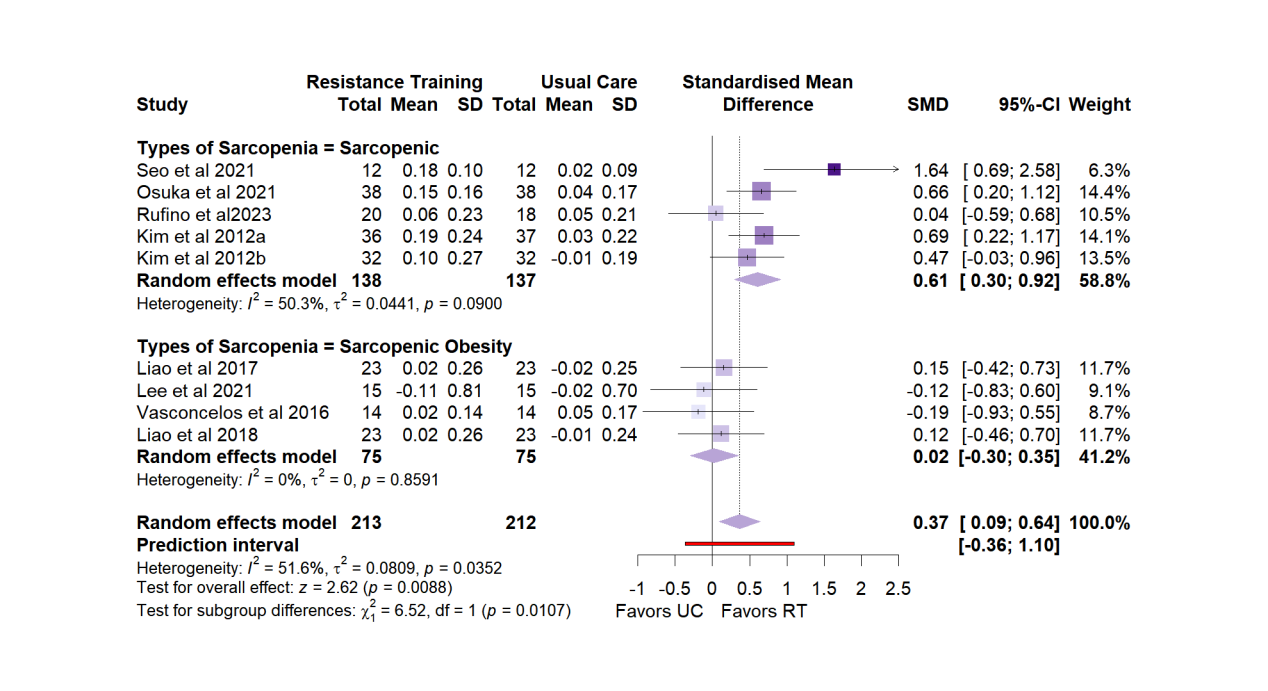


**Figure S4.2.4:** Funnel plot of TUG


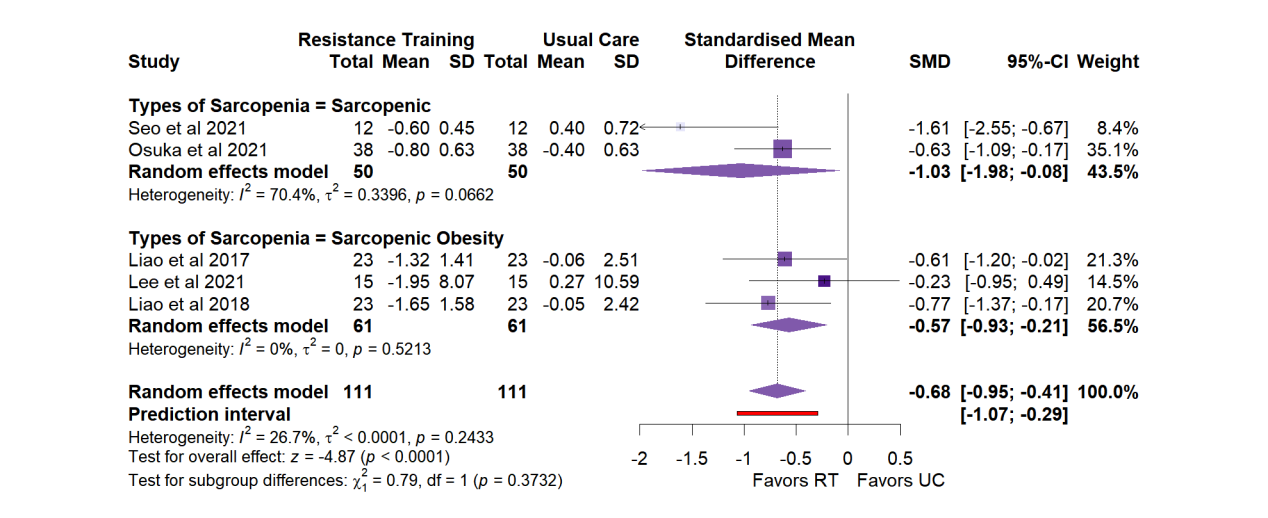


**Figure S4.2.5:** Funnel plot of SMI


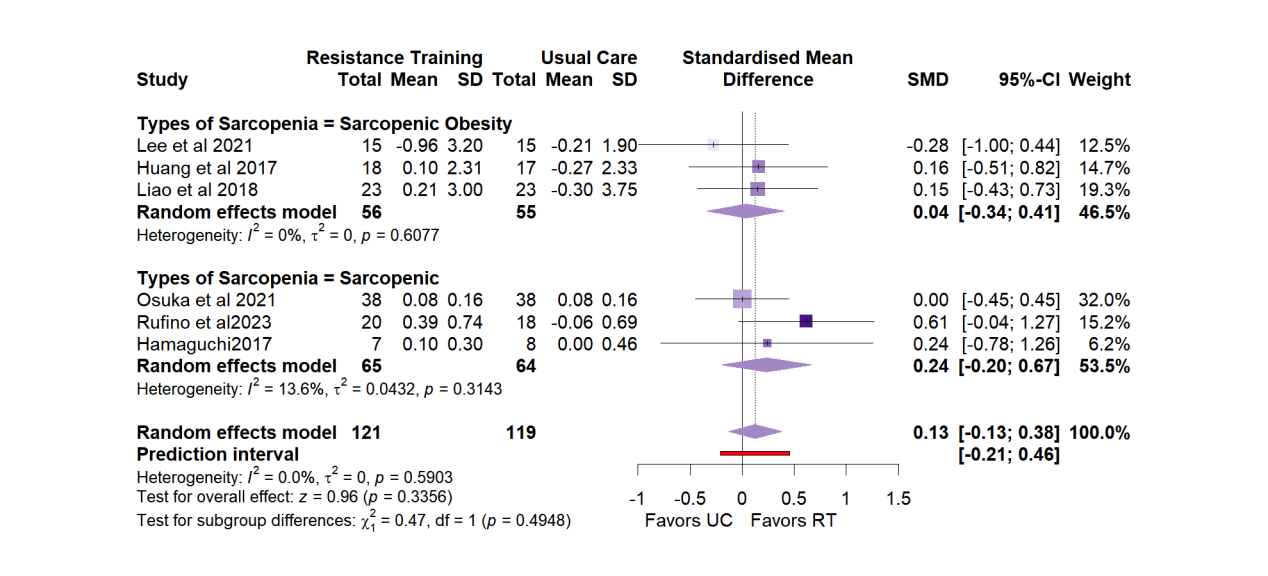


**Appendix 4.3 Subgroup Analysis by type of resistance training**

**Figure S4.3.1:** Funnel plot of Handgrip strength


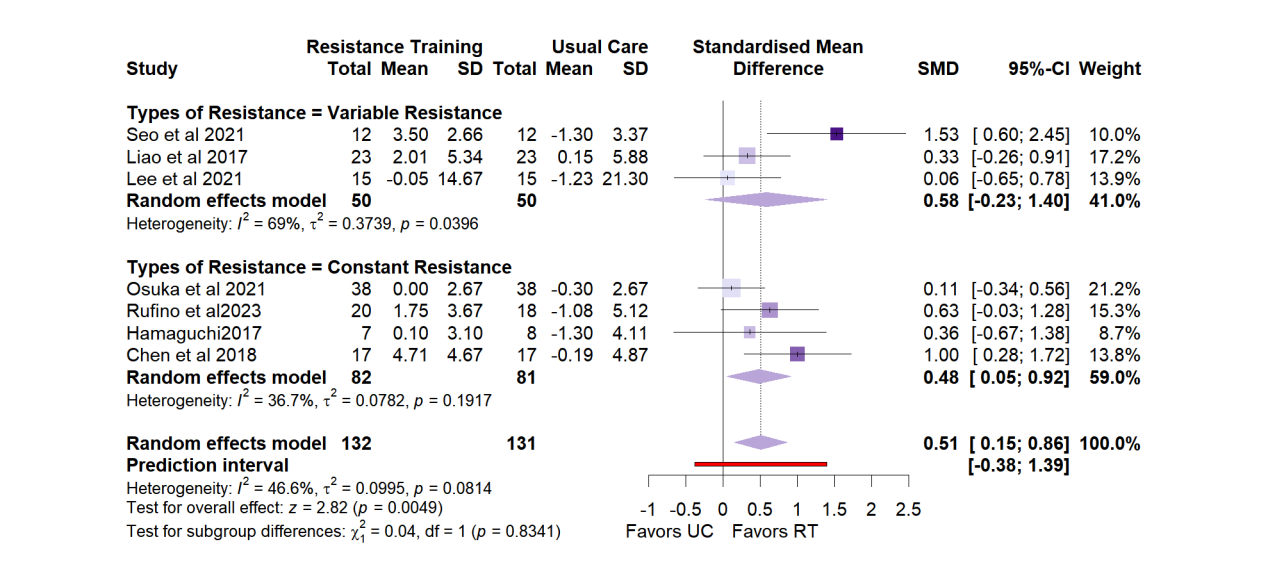


**Figure S4.3.2:** Funnel plot of Knee extension strength


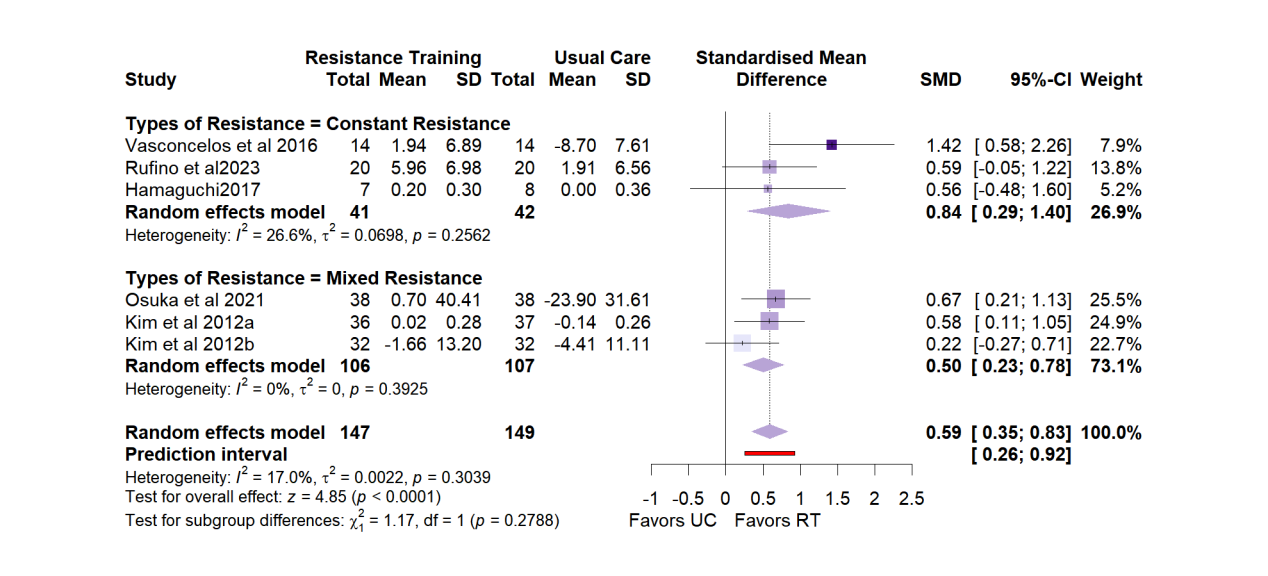


**Figure S4.3.3:** Funnel plot of Gait speed


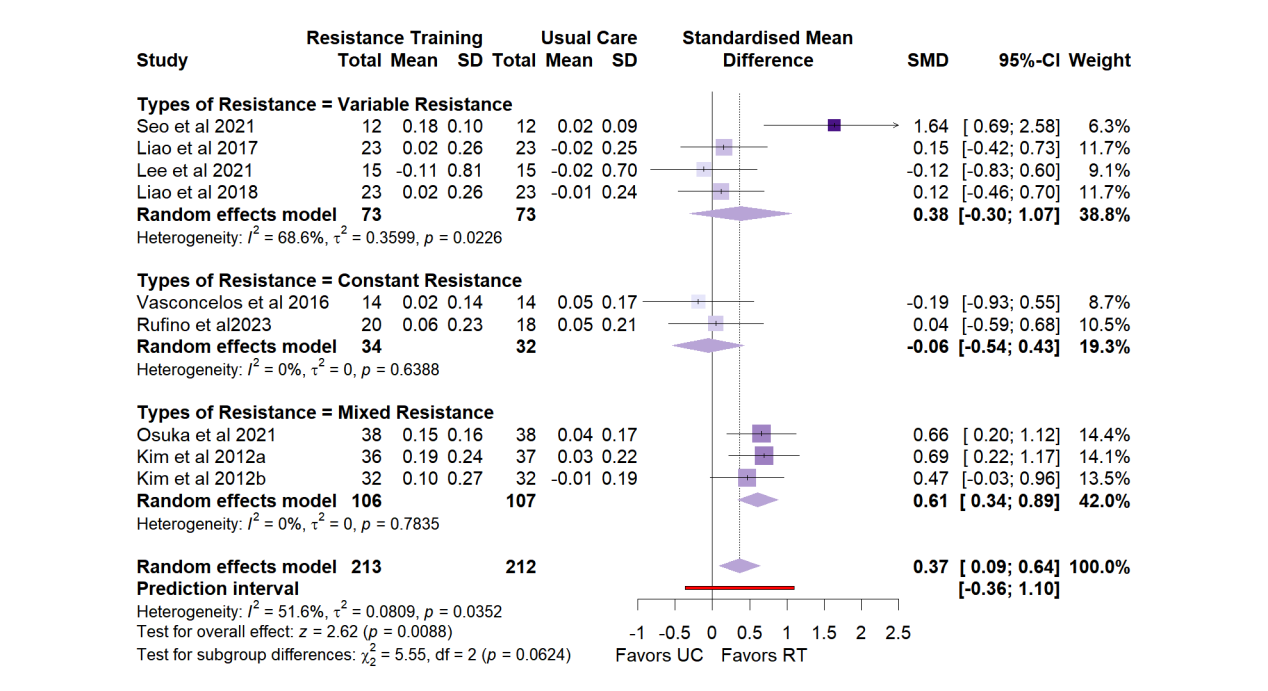


**Figure S4.3.4:** Funnel plot of SMI


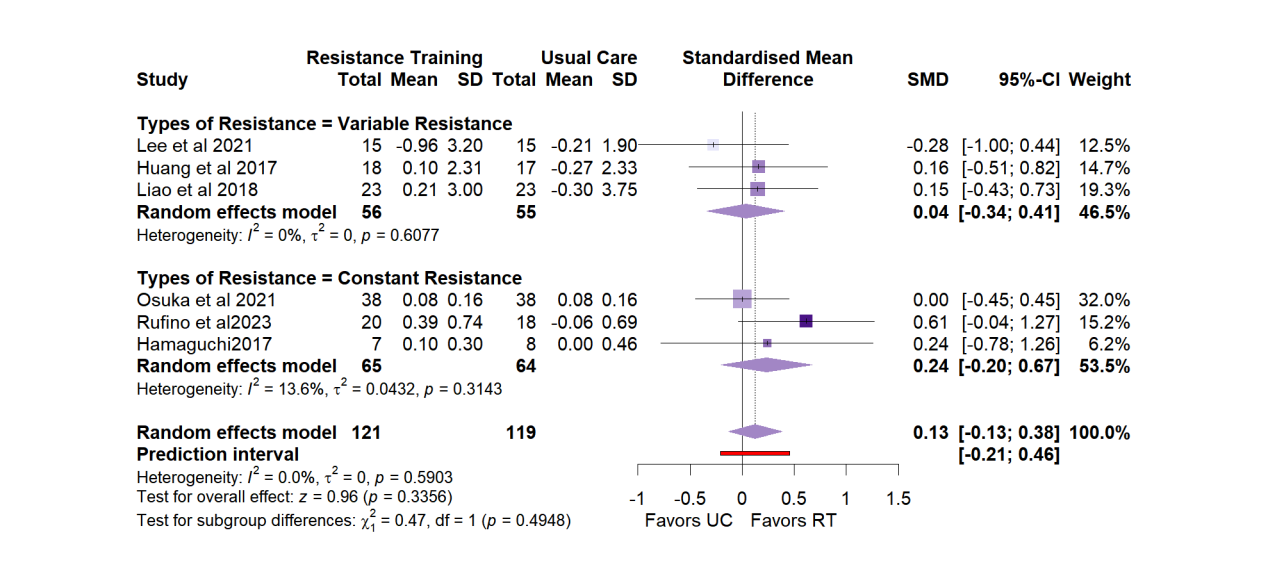


**Appendix 5: Sensitivity analysis**

**Figure S5.1:** Leave-One-Out Sensitivity Analysis for TUG


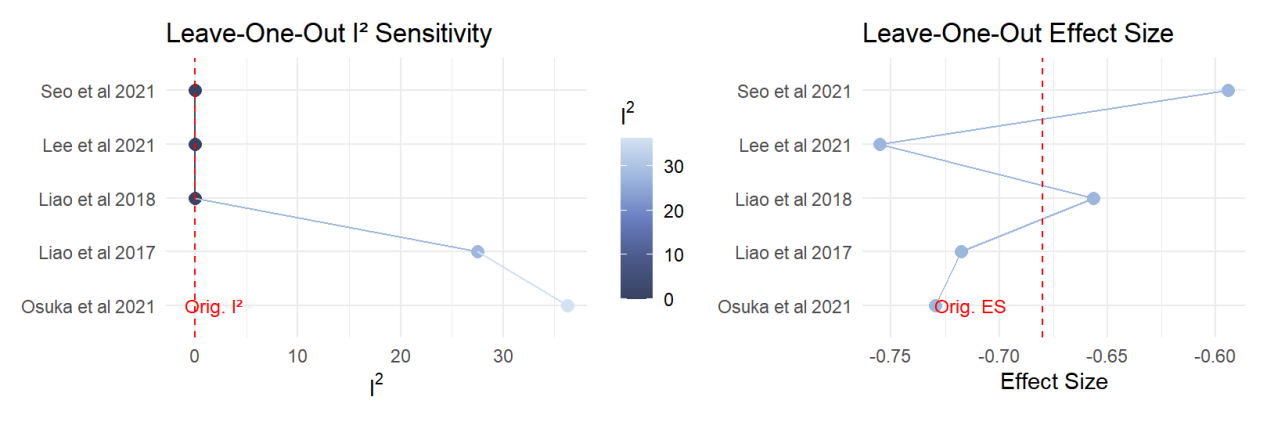


**Figure S5.2:** Leave-One-Out Sensitivity Analysis for CST


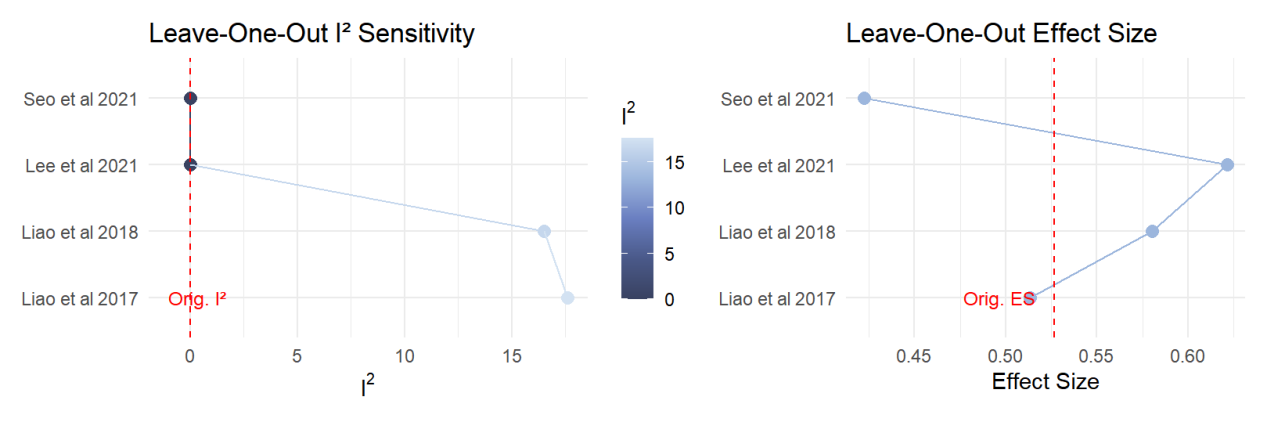


**Figure S5.3:** Leave-One-Out Sensitivity Analysis for SMI


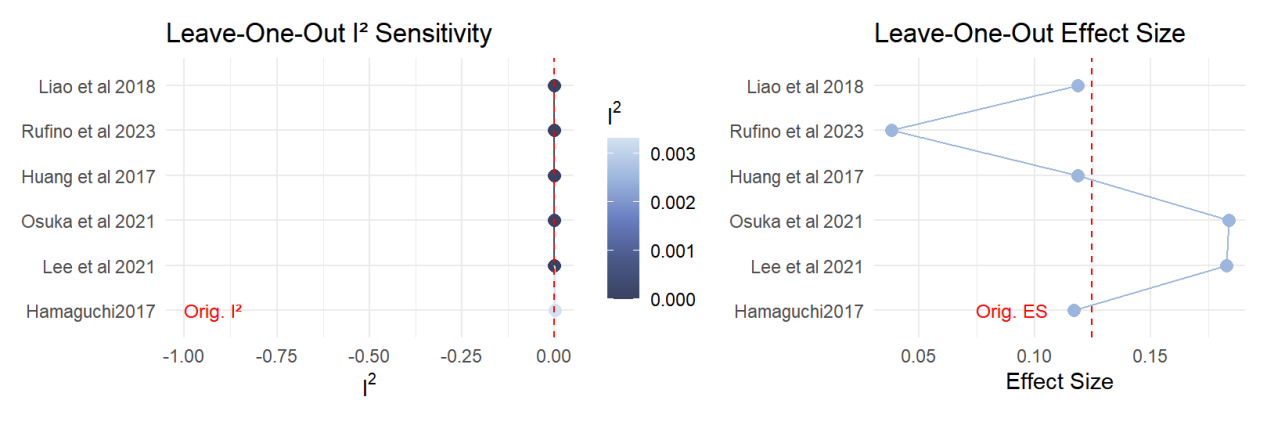

Supplement: Supplementary file 1 [file Data_Sheet_1.docx]
